# Impact of spring festival on pregnancy outcomes in patients undergoing first embryo transfer: a retrospective cohort study

**DOI:** 10.1038/s41598-025-05029-6

**Published:** 2025-07-02

**Authors:** Yuanyuan Yang, Zhaojuan Hou, Huiyu Qiu, Tianli Chang, Nenghui Liu, Donge Liu, Yumei Li, Jing Zhao, Qiong Zhang, Zhongyuan Yao, Fen Tian, Tianli Yang, Yanping Li

**Affiliations:** 1 Reproductive Medicine Center, Xiangya Hospital of Central South University, No.87 Xiangya Road, Changsha, 410008 Hunan P R China; 2 Clinical Research Center for Women’s Reproductive Health in Hunan Province, No.87 Xiangya Road, Changsha, 410008 Hunan P R China

**Keywords:** Culture, Spring festival, Holiday effects, Assisted reproductive technology, Pregnancy outcome, Medical research, Health care, Outcomes research, Health care, Epidemiology, Outcomes research

## Abstract

To evaluate whether the Spring Festival has an effect on the outcomes of patients undergoing their first embryo transfer (ET) via in vitro fertilization/intracytoplasmic sperm injection (IVF/ICSI). We conducted a retrospective cohort study involving 11,625 first ET cycles were performed at a university-affiliated hospital between January 2014 and March 2022. Women who underwent ET during the peri-Spring Festival periods were designated as the Festival Group, while the remainder were categorized as the Non-Festival Group. Multivariate logistic regression and propensity score matching (PSM) were applied to assess the impact of the “Spring Festival Effect” on pregnancy outcomes. Prior to PSM, patients in Festival Group had lower clinical pregnancy rate (44.39% vs. 52.50%, *P* < 0.001), implantation rate (34.41% vs. 40.43%, *P* < 0.001), ongoing pregnancy rate (38.71% vs. 47.15%, *P* < 0.001), and live birth rate (37.33% vs. 44.82%, *P* < 0.001). After controlling for confounders, a similar trend remained in the PSM analysis with matching ratios ranging from 1:1 to 1:4. In the multivariable regression analysis, the Spring Festival was found to be an independent risk factor for poor prognosis. These findings indicated that sociocultural events, represented by the Spring Festival, decreased the chances of IVF/ICSI success, underscoring the importance for clinicians and patients to be more vigilant with healthcare during major social events.

**Supplementary Information:**

The online version contains supplementary material available at 10.1038/s41598-025-05029-6.

## Introduction

The increasing prevalence of infertility among reproductive-aged couples worldwide has been driven by rapid societal development, lifestyle changes, and environmental shifts, with China reporting a notable rise from 12.0 to 18.0% from 2007 to 2020^1^.Since the first infant was conceived with assisted reproductive technology (ART) in 1978, more than 5 million babies have been born through in vitro fertilization and embryo transfer (IVF-ET) around the world^2^. There were over 500 assisted reproductive centers in mainland China by the end of 2019, and the number of ART cycles exceeded 1 million in 2016 and reached 1.15 million in 2017^3^. ART has become increasingly mature, with implantation rates ranging from less than 5.0% per embryo transferred initially to more than 50.0% currently^4^. The overall success rate of ART in China reflects a clinical pregnancy rate of 30.0% and a live birth rate of 28.8%, aligning closely with international levels^3^. Nevertheless, UK statistics revealed that the birth rate per embryo transferred for all IVF patients was only 23% in 2018^5^. Therefore, various factors that may negatively affect success rates are gradually being identified, and researchers are exploring approaches to improve ART outcomes.

The routine procedures of IVF-ET involve controlled ovarian stimulation (COS), oocyte retrieval and sperm collection, IVF to form embryos, and embryo transfer (ET). Given this, the quality of oocyte, sperm, embryo, and the endometrium is essential for the success of ART. Moreover, aspects such as emotional well-being^6,7^stress^8^physical activity^9,10^and even sleep quality^11^also have implications for pregnancy outcomes, in addition to the abovementioned biological concerns. Beyond the intrinsic factors of the infertile couples, external environmental factors, including temperature, light duration and seasonal variation were the most studied^12–14^. Specifically, a significant correlation was found between monthly fertilization rates and the Δ (increase/decrease) in light hours^12^. Sperm concentration was significantly lower in autumn and fast motility was lower in summer than in the other seasons, suggesting seasonal variation^15^.

In contrast to the well-documented effects of natural environmental variation, the potential impact of socio-cultural influences, ranging from beliefs and values to customs and traditions, has been largely overlooked. The Spring Festival is the most significant traditional festival in China and an integral part of Chinese cultural heritage. This annual celebration usually occurs in early February, with preparations and festivities often spanning several weeks before and after the event. Deeply embedded in the daily lives of Chinese people, the festival provides a unique context for exploring how external sociocultural factors may influence human health. On the one hand, during the festival, individuals may transition abruptly from their regular routines to indulgent behaviors, such as overeating, excessive alcohol consumption, disrupted sleep schedules, and heightened emotional states. Type 1 diabetes mellitus (T1DM) patients undergoing multiple daily insulin injections exhibited poorer glycemic control during the Spring Festival compared to those using continuous subcutaneous insulin infusion, suggesting that reduced self-management during the holiday period may negatively impact health^16^. On the other hand, limited medical resources and delays in seeking care might be responsible for high mortality during the festival^17^.

This study sheds light on how socio-cultural events may influence ART outcomes. We hypothesized that both patients and reproductive specialists might be influenced by the festival atmosphere during the Spring Festival, potentially affecting the success of ART. We conducted a retrospective analysis of infertile women who underwent their first ET at our reproductive medicine center between 2014 and 2022. Pregnancy outcomes were compared between those who underwent ET during the “peri-Spring Festival” period and those who underwent the procedure outside of this timeframe.

## Materials and methods

### Ethical approval

This study was a retrospective analysis approved by the ethical committee of Xiangya Hospital of Central South University (CSU) (No. 2022011), and was in accordance with the Helsinki Declaration.

### Data source and study population

**Fig. 1:**
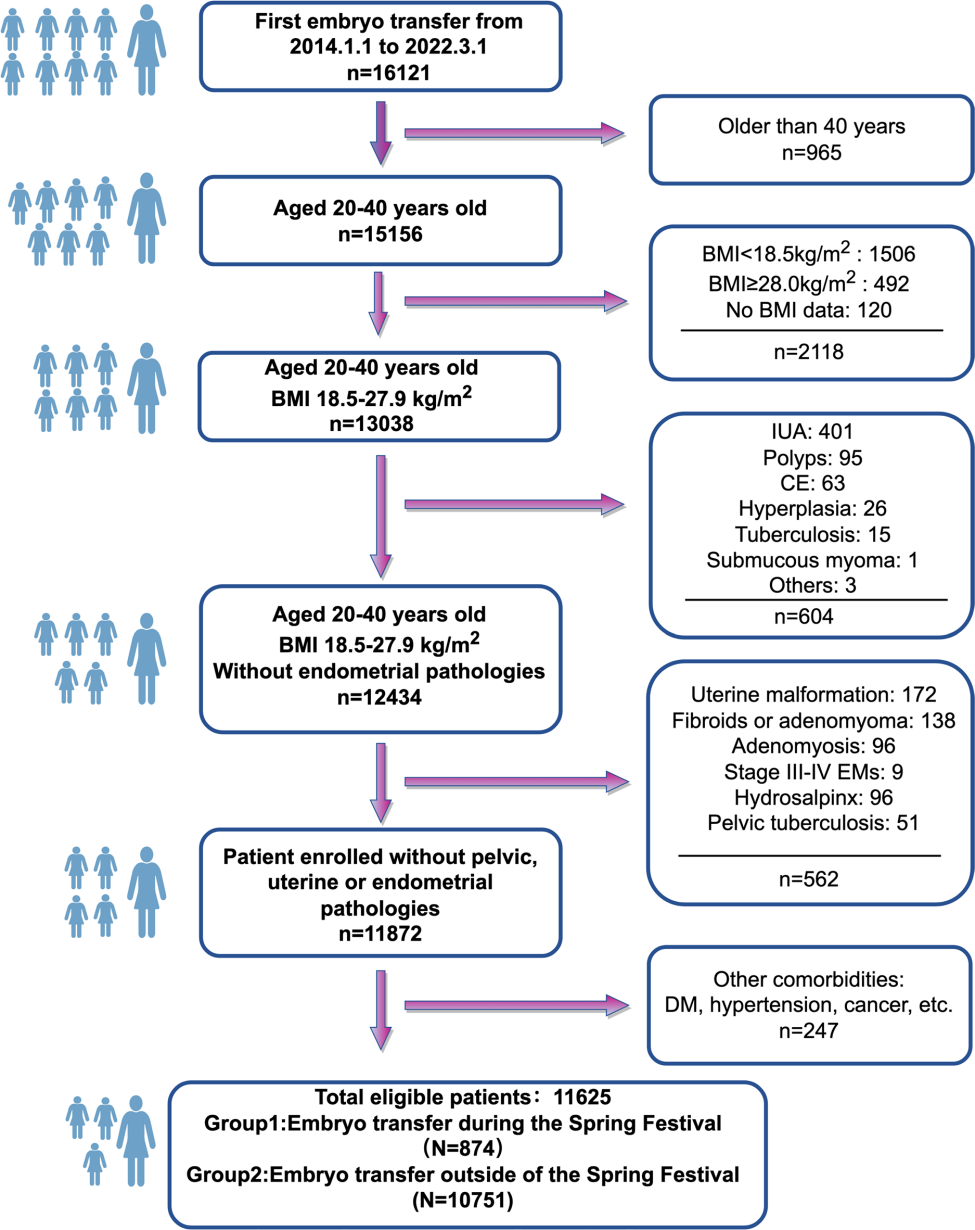
Flowchart showing the patients screened and included in the study.

In this population-based retrospective cohort study, clinical records of 16,121 couples who underwent their first ET between January 1, 2014 and March 1, 2022 at the Reproductive Medicine Center of Xiangya Hospital, CSU, were initially screened. The study design and the inclusion/exclusion criteria are described in Fig. 1. Women aged 20–40 years with a body mass index (BMI) of 18.5–28.0 kg/m^2^ were included. Patients with endometrial abnormalities such as endometrial polyps, endometrial hyperplasia, submucosal fibroids, severe intrauterine adhesions or untreated chronic endometritis were excluded. Patients with hypertension, diabetes, chronic kidney disease, autoimmune disorders or other systemic diseases were also excluded. After applying the inclusion and exclusion criteria, 11,625 patients were included in the final analysis.

The cohort was subdivided into two groups based on the date of ET. The term “peri-Spring Festival” refers to the period spanning three weeks prior to Chinese New Year’s Eve (i.e., Laba Festival) and two weeks after the Lantern Festival. Patients who underwent ETs during the “peri-Spring Festival” period were assigned to the Festival Group, while the remaining cases were designated as the Non-Festival Group.

Demographic data collection included maternal age, BMI, educational level, family residence, occupational status, occupational exposure, infertility duration, type of infertility, gravidity, parity, main indication for ART, anti-Mullerian hormone (AMH) levels, thyroid-stimulating hormone (TSH), serum lipids (triglyceride, total cholesterol, low-density lipoprotein, high-density lipoprotein), fasting blood glucose, fasting insulin, basal antral follicle count (AFC), and basal reproductive hormone levels such as follicle stimulating hormone (FSH), luteinizing hormone (LH), estradiol (E_2_) and testosterone (T).

Cycle-specific variables were also collected, including the type of cycle (fresh and frozen-thawed), method of fertilization [IVF, intracytoplasmic sperm injection (ICSI) and IVF + ICSI], date of ET, endometrial thickness and morphological type measured on the day of ovulation trigger for fresh cycles and on the day of endometrial preparation for frozen-thawed cycles. Embryonic data, such as the stage of embryo development (cleavage or blastocyst), the number and grade of embryos transferred, were collected. In addition, the clinician’s title and gender who performed ET were recorded.

### Clinical management of fresh and frozen ET cycles

For the fresh ET cycle, the standard protocols for COS included the GnRH agonist long protocol (using either short-acting or long-acting preparations), ultra-long protocol, short protocol, and the GnRH antagonist protocol, as described previously^18,19^. Briefly, in the GnRH agonist protocol, COS was initiated once sufficient down-regulation of the pituitary was achieved (serum E_2_ level < 50 pg/ml and a maximum follicle diameter < 10 mm with no ovarian cysts). In the GnRH short protocol and GnRH antagonist protocol, COS was initiated on day 2 or day 3 or the menstrual cycle. The initial dose of exogenous gonadotropins (Gn) ranged from 112.5 to 300.0 IU/day, depending on the individuals’ body weight and ovarian reserve. The Gn dosage was then adjusted based on follicular development assessed by transvaginal ultrasonography and serum E_2_ levels. When the follicles reached a size indicating maturity (dominant follicles ≥ 18 mm in diameter), human chorionic gonadotrophin (hCG) or a GnRH agonist was used to simulate the LH surge and trigger final oocyte maturation. Oocytes were usually retrieved 36 h after triggering and fertilized by IVF or ICSI 4–6 h after retrieval. Fresh embryos were transferred on day 3 or day 5 after fertilization.

For frozen-thawed embryo transfer (FET) cycles, various protocols were used for endometrial preparation at our center, including natural cycles (also encompassing stimulated natural cycles), hormone replacement therapy (HRT) cycles, and GnRH agonist + HRT cycles. Among these, natural and HRT cycles were the most commonly applied^20,21^. Natural FET cycles were recommended for women with regular ovulatory menstrual cycles, and ovulation was monitored by transvaginal ultrasonography starting from day 10 of the menstrual cycle until follicular rupture. For HRT cycles, estradiol valerate was initiated on day 3 of the menstrual cycle and continued for at least 12 days. Progesterone was added to induce endometrial transformation when the endometrial thickness exceeded 7 mm. In natural FET cycles, cleavage-stage embryos or blastocysts were transferred on day 3 or day 5 after ovulation, respectively. In HRT cycles, embryos were transferred on the corresponding days after the initiation of progesterone supplementation.

Vaginal micronized progesterone (Utrogestan; Besins-Iscovesco Pharmaceuticals, Paris, France) at 600 mg/day and oral progesterone capsules (Qining; Aisheng, Hangzhou, China) at 200 mg/day were administrated for luteal support. Serum hCG levels were measured 12 days after ET, and transvaginal ultrasound was conducted 28 days after transfer to confirm clinical pregnancy.

### Pregnancy outcomes

The primary outcomes included clinical pregnancy rate and live birth rate. Clinical pregnancy refers to the number of patients with intrauterine pregnancy per ET cycle. Live birth was defined as the delivery of any viable neonate who was 28 weeks of gestation or older.

The secondary outcome were positive hCG rate, biochemical pregnancy rate, implantation rate (IR), ectopic pregnancy rate, early miscarriage rate, late miscarriage rate, ongoing pregnancy rate, preterm birth rate and term birth rate. We defined a positive pregnancy test as β-hCG > 10IU/L. Biochemical pregnancy was defined as a very early spontaneous abortion after a positive pregnancy had been determined and with a fall in serum β-hCG concentration before the ultrasonic detection of gestational sacs. IR refers to the number of gestational sacs observed divided by the number of embryos transferred. Ectopic pregnancy was defined as any extrauterine implantation, including heterotopic pregnancies.

Ongoing pregnancy was defined as a sustained pregnancy beyond 12 weeks of gestation. Early miscarriage was classified as occurring before 12 weeks, while late miscarriage was defined as occurring between 12 and 28 weeks. Preterm birth was defined as the delivery between 28 weeks and less than 37 weeks of gestation. Term birth was defined as delivery at 37 weeks of gestation or beyond.

### Statistical analysis

All statistical analyses were performed using SPSS (version 25.0) and R (version 4.3.3). The Kolmogorov–Smirnov test was used to assess the normality of continuous variables. If the data followed a normal distribution, they were expressed as mean ± standard deviation (SD); otherwise, they were described as median and interquartile range (IQR; 25th and 75th percentiles). Categorical variables were presented as frequencies (n) and percentages (%).

For comparison between the two groups, the independent t-test and Wilcoxon rank-sum test were used to determine significance for continuous variables as appropriate, and Pearson’s chi-square test was conducted to evaluate differences in categorical variables. Unadjusted and adjusted odds ratios (ORs) with 95% confidence intervals (CIs) were reported for reproductive outcomes.

To minimize potential baseline differences and reduce selection bias between the Festival Group and Non-Festival Group, a propensity score matching (PSM) analysis was performed using the MatchIt package in R based on the methodological and reporting guidelines proposed by Yao et al.^22^. Matched outcomes were analyzed and presented in accordance with these guidelines.

Propensity scores were estimated via logistic regression using covariates that were selected with the aim of achieving adequate matching with minimal model complexity. Specifically, variables that showed significant differences between groups in the unmatched cohort (based on independent t-tests or chi-square tests), as well as those deemed clinically relevant based on prior knowledge or expert opinion, were incorporated into the model (see Supplementary Table 1 for details). No missing values existed in the covariates for propensity score estimation.

Nearest-neighbor matching without replacement was performed using four different matching ratios: 1:1, 1:2, 1:3, and 1:4, with a caliper width of 0.02 on the logit of the propensity score. Baseline characteristics between the matched groups were compared to assess the quality of the match. Standardized mean differences (SMDs) were calculated for all covariates after matching, with an SMD < 0.1 considered indicative of adequate balance.

Prior to multivariate analysis, univariate logistic regression was performed for each outcome variable to identify potential covariates (see Supplementary Tables 3–6). Binary logistic regression models were then applied to evaluate the independent effect of the Spring Festival period on positive β-hCG, intrauterine pregnancy, ongoing pregnancy, and live birth, respectively. For example, for the outcome of positive β-hCG, the model was adjusted for maternal age at embryo transfer, education level, current career status, duration of infertility, type of infertility, main indication for ART, basal FSH, basal LH, fertilization method, endometrial thickness, endometrial type, stage of embryo development and number of embryos transferred. Statistical significance was assigned to two-sided P values below 0.05 (*p* < 0.05).

## Results

### Characteristics of the study population before PSM and after PSM

A total of 16,121 first ET cycles were performed from January 1 2014 to March 1 2022, of which 11,625 cycles (corresponding to 11,625 patients) met the inclusion criteria and were included in the final analysis (Fig. 1). We conducted 1:1, 1:2, 1:3, and 1:4 PSM analyses; in this part, only the results from the 1:4 matching are presented, while results from other matching ratios are provided in Supplementary Tables 1 and 2. The Festival group consisted of 874 participants before matching and 860 participants after PSM. The Non-Festival group comprised 10,751 patients before matching and 3,354 after PSM. The demographics and clinical parameters before and after matching are summarized in Table 1.

**Table 1:** Baseline characteristics of the study population before and after PSM.

	Full cohort			1:4 Propensity-matched cohort^b^			
Characteristics	Festival group (*N* = 874)	Non-festival group (*N* = 10751)	*P* Value	Festival group (*N* = 860)	Non-festival group (*N* = 3354)	*P* value	SMD
Maternal age at embryo transfer (years; mean (SD))	30.65 ± 4.23	30.42 ± 4.17	0.124	30.60 ± 4.22	30.61 ± 4.10	0.983	0.001
Maternal BMI (kg/m2; mean (SD))	21.79 ± 2.55	22.12 ± 2.34	< 0.001	21.81 ± 2.55	21.83 ± 2.28	0.828	0.009
Education, n (%)			0.002			0.227	0.069
Primary school and below	42(4.80%)	611(5.70%)		42 (4.88%)	182 (5.43%)		
Secondary and High Schools	485(55.50%) ^a^	6509(60.50%)		479 (55.70%)	1961 (58.47%)		
College and above	347(39.70%) ^a^	3631(33.80%)		339 (39.42%)	1221 (36.10%)		
Family residence, n (%)			0.023			0.127	0.077
Changsha city	91(10.40%) ^a^	861(8.00%)		90 (10.47%)	292 (8.70%)		
Outside Changsha and within Hunan Province	520(59.50%)	6361(59.20%)		512 (59.53%)	1962 (58.50%)		
Outside Hunan Province	263(30.10%)	3529(32.80%)		258 (30.00%)	1100 (32.80%)		
Occupational status, n (%)			0.010			0.287	0.075
Employed	357(40.80%) ^a^	3907(36.30%)		352 (40.93%)	1290 (38.46%)		
Self-employed	100(11.40%) ^a^	1601(14.90%)		99 (11.51%)	459 (13.69%)		
Freelance	126(14.40%)	1626(15.10%)		126 (14.65%)	515 (15.35%)		
Housewife	291(33.30%)	3617(33.60%)		283 (32.91%)	1090 (32.50%)		
Occupational exposure, n (%)			0.937			0.595	0.020
‘Unexposed’ occupation	808(92.40%)	9947(92.50%)		794 (92.33%)	3078 (91.77%)		
‘Exposed’ occupation	66(7.60%)	804(7.50%)		66 (7.67%)	276 (8.23%)		
Duration of infertility (years; median (interquartile range))	3.71 ± 2.85	4.02 ± 3.05	0.005	3.73 ± 2.86	3.76 ± 2.91	0.737	0.013
Type of infertility, n(%)			0.839			0.734	0.013
Primary infertility	411(47.0%)	5094(47.40%)		403 (46.86%)	1550 (46.21%)		
Secondary infertility	463(53.0%)	5657(52.60%)		457 (53.14%)	1804 (53.79%)		
Gravidity (n; median (interquartile range))	1(0,2)	1(0,2)	0.816	1 (0,2)	1 (0,2)	0.287	0.054
Parity (n; median (interquartile range))	0(0,0)	0(0,0)	0.375	0 (0,0)	0 (0,0)	0.170	0.045
Main indication for ART, n(%)			0.001			0.652	0.078
Tubal factor	561(64.20%) ^a^	7259(67.50%)		557 (64.77%)	2184 (65.12%)		
Ovulation disorders	78(8.90%)	963(9.00%)		78 (9.07%)	322 (9.60%)		
Endometriosis	48(5.50%)	457(4.30%)		46 (5.35%)	178 (5.31%)		
DOR	32(3.70%)	378(3.50%)		32 (3.72%)	128 (3.82%)		
Male factor	117(13.40%)	1415(13.20%)		114 (13.26%)	422 (12.58%)		
RSA	10(1.10%)	84(0.80%)		3 (0.35%)	1 (0.03%)		
Chromosomal abnormality	20(2.30%)	174(1.60%)		10 (1.16%)	38 (1.13%)		
Unexplained infertility	8(0.90%) ^a^	21(0.20%)		20 (2.33%)	81 (2.42%)		
AMH, ng/ml; median (IQR)	3.43(2.11,5.48)	3.54(2.00,5.81)	0.231	3.34 (1.94,5.37)	3.64 (2.07,6.07)	0.309	0.104
Basal FSH, mIU/ml; median (IQR)	6.21(5.20,7.50)	6.30(5.30,7.50)	0.378	6.30 (5.30,7.72)	6.36 (5.28,7.55)	0.338	0.045
Basal LH, mIU/ml; median (IQR)	4.80(3.60,6.30)	4.90(3.53,6.61)	0.220	4.79 (3.69,6.40)	5.11 (3.70,7.00)	0.168	0.097
Basal estradiol, pg/ml; median (IQR)	34.51(26.17,45.26)	34.00(25.00,44.40)	0.112	35.68 (27.00,47.55)	35.17 (26.53,45.48)	0.247	0.042
Testosterone, ng/mL; median (IQR)	0.25(0.16,0.34)	0.24(0.16,0.33)	0.523	0.26 (0.17,0.35)	0.25 (0.17,0.35)	0.576	0.025
AFC, n (%)			0.118			0.247	0.079
1–6	83(9.50%)	1220(11.30%)		81 (9.42%)	378 (11.27%)		
7–12	257(29.40%)	3386(31.50%)		251 (29.19%)	1015 (30.26%)		
13–24	513(58.70%) ^a^	5918(55.00%)		507 (58.95%)	1866 (55.64%)		
> 24	21(2.40%)	227(2.10%)		21 (2.44%)	95 (2.83%)		
Thyroid-stimulating hormone, uIU/ml; median (IQR)	2.17(1.50,3.07)	2.17(1.51,3.06)	0.737	2.11 (1.48,2.96)	2.24 (1.55,3.06)	0.825	0.006
Serum triglyceride (mmol/L; median (IQR))	0.99(0.75,1.46)	1.06(0.78,1.48)	0.291	1.04 (0.79,1.53)	1.08 (0.81,1.47)	0.051	0.029
Total cholesterol (mmol/L; median (IQR))	4.61(4.12,5.13)	4.62(4.11,5.19)	0.922	4.59 (4.10,5.11)	4.66 (4.11,5.20)	0.692	0.010
Low-density lipoprotein cholesterol (mmol/L; median (IQR))	2.74(2.31,3.16)	2.71(2.29,3.19)	0.835	2.76 (2.34,3.17)	2.76 (2.33,3.21)	0.609	0.009
High-density lipoprotein cholesterol (mmol/L; median (IQR))	1.40(1.20,1.65)	1.41(1.21,1.64)	0.376	1.34 (1.15,1.59)	1.40 (1.21,1.61)	0.139	0.071
Dyslipidemia, (%, n)	38.20% (247/646)	39.40% (3082/7815)	0.548	38.60% (245/635)	38.70% (930/2401)	0.945	0.003
Fasting blood glucose (mmol/L; median (IQR))	5.27(5.00,5.56)	5.29(5.03,5.56)	0.128	5.23 (4.94,5.53)	5.26 (5.01,5.55)	0.390	0.045
Fasting insulin (uU/mL; median (IQR))	9.00(6.55,12.90)	9.58(6.92,13.18)	0.007	8.98 (6.39,13.36)	9.34 (6.73,13.03)	0.381	0.028
HOMA-IR, % (n/N)			0.051			0.651	0.020
< 2.5	62.70% (399/636)	58.80% (4588/7806)		62.52% (392/627)	61.50% (1470/2389)		
≥ 2.5	37.30% (237/636)	41.20% (3218/7806)		37.48% (235/627)	38.50% (919/2389)		
Type of cycle, % (n/N)			< 0.001			0.357	0.035
Cycle with fresh embryo transfer	61.9% (541/874)	72.4% (7786/10751)		62.44% (537/860)	64.13% (2151/3354)		
Cycle with frozen-thawed embryo transfer	38.1% (333/874)	27.6% (2965/10751)		37.56% (323/860)	35.87% (1203/3354)		
Fertilization method, n (%)			0.278			0.466	0.048
IVF	636(72.80%)	7842(72.90%)		628 (73.02%)	2431 (72.48%)		
ICSI	187(21.40%)	2149(12.00%)		182 (21.16%)	689 (20.54%)		
IVF + ICSI	51(5.80%)	760(7.10%)		50 (5.81%)	234 (6.98%)		
Endometrial thickness (mm; mean (SD))	10.47 ± 2.18	10.59 ± 2.14	0.126	10.48 ± 2.19	10.47 ± 2.15	0.947	0.005
Endometrial type, n (%)			0.011			0.706	0.032
A	323(37.00%) ^a^	4527(42.10%)		322 (37.44%)	1276 (38.04%)		
B	483(55.30%) ^a^	5496(51.10%)		474 (55.12%)	1805 (53.82%)		
C	68(7.80%)	728(6.80%)		64 (7.44%)	273 (8.14%)		
Stage of embryo development, n (%)			< 0.001			0.816	0.028
Cleavage stage embryos	1313 (86.73%)	17,633 (91.27%)		1305 (87.23%)	5116 (87.01%)		
Blastocyst stage embryos	201 (13.27%)	1686 (8.73%)		191 (12.17%)	764 (12.99%)		
No. of embryos transferred (n; mean (SD))	1.73 ± 0.44	1.80 ± 0.40	< 0.001	1.74 ± 0.44	1.75 ± 0.43	0.411	0.031
Good-quality embryos transferred rate, % (n/N)	88.84% (1345/1514)	92.10% (17793/19319)	< 0.001	89.30% (1336/1496)	89.54% (5265/5880)	0.790	0.029
Clinician’ title, n (%)			0.057			0.832	0.008
Associate chief physician and above	692(79.20%)	8791(81.80%)		688 (80.00%)	2694 (80.32%)		
Attending physician	182(20.80%)	1960(18.20%)		172 (20.00%)	660 (19.68%)		
Clinician’ gender, n (%)			0.080			0.658	0.017
Female	614(70.30%)	7847(73.00%)		607 (70.58%)	2393 (71.35%)		
Male	260(29.70%)	2904(27.00%)		253 (29.42%)	961 (28.65%)		

* PSM* Propensity Score Matching, *SD* Standard Deviation, *SMD* Standardized Mean Differences, *BMI* Body Mass Index, *ART* Assisted Reproductive Technology, *DOR* Diminished Ovarian Reserve, *RSA* Recurrent Spontaneous Abortion, *AMH* anti-Mullerian Hormone, *FSH* Follicle Stimulating Hormone, *LH* Luteinizing Hormone, *AFC* Antral Follicle Count, *HOMA-IR* Homeostatic Model Assessment for Insulin Resistance, *IVF* In Vitro Fertilization, *ICSI* Intracytoplasmic Sperm Injection, *IQR* Interquartile Range.

^a^ Festival Group vs. Non-Festival Group: *P* < 0.05.

^b^1:4 PSM was matched for maternal age at ET, maternal BMI, duration of infertility, main indication for ART, type of cycle, endometrial type, stage of embryo development, number of embryos transferred, good-quality embryos transferred, clinician’s title and clinician’s gender.

Before PSM, the Festival Group had a lower BMI, a higher proportion of women with a college education or above, a greater proportion residing in Changsha City, and more patients who were employed compared to the Non-Festival Group (*P* < 0.05 for all). Additionally, the Festival Group exhibited a shorter mean duration of infertility, lower proportion of tubal factor infertility as the main indication for ART, lower fasting insulin levels, and fewer cycles involving fresh ET. All of these differences reached statistical significance (*P* < 0.05). The Festival Group also showed a lower proportion of patients with type A endometrium and a higher proportion with type B endometrium (*P* = 0.011). Additionally, more blastocyst-stage embryos were transferred in the Festival group compared to the Non-Festival group (13.27% vs. 8.73%, *P* < 0.001). The mean number of embryos transferred per cycle was slightly lower (1.73 ± 0.44 vs. 1.80 ± 0.40, *P* < 0.001), and the rate of good-quality embryo transfer was significantly lower (88.84% vs. 92.10%, *P* < 0.001). Other parameters including maternal age at ET, occupational exposure, type of infertility, gravidity, parity, AMH, reproductive hormones, AFC, TSH, serum lipids, fasting blood glucose, fertilization method, endometrial thickness, clinician’s title and clinician’s gender were comparable between the two groups (*P* > 0.05).

We performed a 1:4 PSM based on maternal age, BMI, duration of infertility, endometrial type, number and quality of embryos transferred, main indication for ART, type of cycle, stage of embryo development, and the title and gender of the embryo transfer physician. After 1:4 PSM, 860 participants remained in the Festival Group and 3,354 in the Non-Festival Group. Except for AMH (SMD = 0.104, *P* = 0.309), all other covariates had SMDs < 0.1 and *P* > 0.05 after matching, indicating adequate balance between groups. In addition, the baseline characteristics after 1:1 to 1:3 PSM, which effectively balanced the differences in confounding factors between the two groups, are presented in Supplementary Table 1.

### Clinical outcomes of participants before and after PSM

Among the full cohort, both clinical pregnancy and live birth rate were significantly lower in the Festival Group than in the Non-Festival Group (clinical pregnancy: 44.39% vs. 52.50%; live birth: 37.33% vs. 44.82%; *P* < 0.001 for both comparisons, Table 2). Among secondary outcomes, the Festival Group also showed significantly lower positive β-hCG rate (55.03% vs. 62.56%), implantation rate (34.41% vs. 40.43%), ongoing pregnancy rate (38.71% vs. 47.15%) and term birth rate (29.72% vs. 35.94%) (all *P* < 0.001). There were no significant differences in biochemical pregnancy rate, ectopic pregnancy rate, miscarriage rate (including early and late miscarriage), preterm birth rate and the proportion of missing transfer outcomes (all *P* > 0.05).

**Table 2:** Pregnancy outcomes of the study population before and after PSM.

Variables	Full cohort			1:4 propensity-matched cohort^a^		
	Festival group (*N* = 874)	Non-festival group (*N* = 10751)	*P* value	Festival group (*N* = 860)	Non-festival group (*N* = 3354)	*P* value
Primary outcome						
Clinical pregnancy rate	44.39% (388/874)	52.50% (5645/10751)	< 0.001	44.53% (383/860)	50.98% (1710/3354)	0.001
Live birth rate	37.33% (324/868)	44.82% (4774/10652)	< 0.001	37.50% (320/853)	44.21% (1471/3327)	< 0.001
Secondary outcome						
Positive β-hCG rate	55.03% (481/874)	62.56% (6726/10751)	< 0.001	55.12% (474/860)	61.87% (2075/3354)	< 0.001
Biochemical pregnancy rate	9.20% (80/874)	8.50% (909/10751)	0.477	9.07% (78/860)	9.27% (311/3354)	0.855
Implantation rate	34.41% (521/1514)	40.43% (7811/19319)	< 0.001	34.49% (516/1496)	40.00% (2352/5880)	< 0.001
Missing data on transfer outcome	0.68% (6/874)	0.92% (99/10751)	0.485	0.81% (7/860))	0.80% (27/3354)	0.979
Ectopic pregnancy rate	4.12% (16/388)	3.35% (189/5645)	0.415	4.18% (16/383)	3.39% (58/1710)	0.452
Miscarriage rate	14.95% (58/388)	13.43% (758/5645)	0.397	14.88% (57/383)	12.16% (208/1710)	0.148
Early miscarriage rate	11.86% (46/388)	9.23% (521/5645)	0.086	11.75% (45/383)	8.89% (152/1710)	0.083
Late miscarriage rate	3.09% (12/388)	4.20% (237/5645)	0.290	3.13% (12/383)	3.27% (56/1710)	0.888
Ongoing pregnancy rate	38.71% (336/868)	47.15% (5022/10652)	< 0.001	38.92% (332/853)	46.00% (1531/3327)	< 0.001
Preterm birth rate	7.60% (66/868)	8.97% (956/10652)	0.172	7.62% (65/853)	9.30% (309/3327)	0.128
Term birth rate	29.72% (258/868)	35.94% (3828/10652)	< 0.001	29.90% (255/853)	35.00% (1166/3327)	0.005

*PSM* Propensity Score Matching, *hCG* human chorionic gonadotropin.

^a^1:4 PSM was adjusted for maternal age at ET, duration of infertility, main indication for ART, type of cycle, endometrial type, stage of embryo development, number of embryos transferred, good-quality embryos transferred, clinician’s title, clinician’s gender.

After 1:4 PSM, the clinical pregnancy rate (44.53% vs. 50.98%, *P* = 0.001), live birth rate (37.50% vs. 44.20%, *P* < 0.001), positive β-hCG rate (55.12% vs. 61.87%, *P* < 0.001), implantation rate (34.49% vs. 40.00%, *P* < 0.001), ongoing pregnancy rate (38.90% vs. 46.00%, *P* < 0.001) and term birth rate (29.90% vs. 35.00%, *P* = 0.005) were significantly lower in the Festival Group compared to the Non-Festival Group. A similar trend was observed in the 1:1 to 1:3 PSM analyses (Supplementary Table 2).

### Effects of “Spring Festival” period on reproductive outcomes after first ET

To investigate whether ET during the Spring Festival period independently affects reproductive outcomes, we conducted binary logistic regression analysis for key reproductive endpoints. After adjusting for relevant confounders (see Supplementary Tables 3–6 for details), ET during the Spring Festival remained significantly associated with worse outcomes, including reduced rates of positive β-hCG (adjusted OR 0.729, 95% CI: 0.631–0.841), clinical pregnancy (adjusted OR 0.717, 95% CI: 0.622–0.827), ongoing pregnancy (adjusted OR 0.708, 95% CI: 0.612–0.819), and live birth (adjusted OR 0.731, 95% CI: 0.631–0.847) (Table 3). The adjusted ORs suggest that undergoing ET during the Spring Festival period is associated with a 27–29% decrease in the likelihood of successful reproductive outcomes. Notably, the specific covariates included in each model differed by outcome and are detailed in Supplementary Tables 3–6.

**Table 3:** Effects of “spring festival” period on reproductive outcomes after first ET.

Variable	OR for positive β-hCG		OR for clinical pregnancy		OR for ongoing pregnancy		OR for live birth	
	Crude OR (95% CI)	Adjusted OR (95% CI) ^a^	Crude OR (95% CI)	Adjusted OR (95% CI) ^a^	Crude OR (95% CI)	Adjusted OR (95% CI) ^a^	Crude OR (95% CI)	Adjusted OR (95% CI) ^a^
Non-festival group	Reference							
Festival group	0.732 (0.637,0.842)	0.729 (0.631,0.841)	0.722 (0.629,0.830)	0.717 (0.622,0.827)	0.712 (0.618,0.820)	0.708 (0.612,0.819)	0.736 (0.638,0.849)	0.731 (0.631,0.847)

*ET* embryo transfer, *OR* odds ratio, *CI* confidence interval, *hCG* human chorionic gonadotropin.

^a^ The model was adjusted for maternal age at embryo transfer, education, occupational status, duration of infertility, type of infertility, main indication for ART, basal FSH, basal LH, fertilization, endometrial thickness, endometrial type, stage of embryo development and number of embryos transferred.

### Evaluating the impact of other holidays on ET outcomes

To investigate whether other national holidays exert similar effects on first ET outcomes as the Spring Festival, we compared baseline characteristics and pregnancy outcomes between transfers performed during the National Day of the People’s Republic of China (October 1 to October 7) versus Non-National Day periods, and the International Workers’ Day (i.e., Labour Day, May 1 to May 5) versus Non-Labour Day periods.

As shown in Supplementary Tables 7, 196 cycles involved ETs during the National Day period, compared to 11,429 cycles performed during Non-National Day periods. Baseline comparisons revealed statistically significant differences in patients’ fasting blood glucose levels and the title of the clinicians performing ET between the two groups (*P* = 0.037 and *P* = 0.024, respectively), while no other baseline characteristics showed significant differences (all *P* > 0.05). Regarding clinical outcomes, no statistically significant differences were observed between the two groups, except for a notably lower preterm birth rate in the National Day Group (3.59% vs. 8.96%, *P* = 0.009) (see Supplementary Table 8).

Similarly, among the entire study population, 108 cycles involved ETs during the Labour Day, while 11,517 cycles were performed outside this period. Baseline comparisons between the two groups revealed statistically significant differences in gravidity, AMH levels, cycle type, good-quality embryos transferred rate, and the gender of the ET physician (see Supplementary Table 7). However, no statistically significant differences were observed in clinical outcomes, including clinical pregnancy rate, positive β-hCG rate, implantation rate, miscarriage rate, and live birth rate (all *P* > 0.05) (see Supplementary Table 8).

To assess whether embryo transfers during the National Day holiday influence pregnancy outcomes, we conducted multivariate logistic regression analyses for various reproductive endpoints. After adjusting for potential confounders, the National Day period was significantly associated with a reduced risk of preterm birth (adjusted OR 0.375, 95% CI: 0.175–0.802, Supplementary Fig. 1d), while no significant associations were observed for other outcomes. As shown in Supplementary Fig. 2, after adjusting for relevant confounders, embryo transfer during the Labour Day period was not significantly associated with any pregnancy outcome, including β-hCG positivity, clinical pregnancy, ongoing pregnancy, or live birth.

## Discussion

Our study focused on the impact of socio-cultural events on the pregnancy outcomes of IVF women undergoing their initial ET procedures. Both PSM and traditional multivariable-adjusted regression analysis confirmed a statistically significant and robust association between the “Spring Festival Effect” and reproductive outcomes. This large study reveals an intriguing phenomenon: patients undergoing ET during the “peri-Spring Festival” have less favorable clinical outcomes compared to those treated on Non-Spring Festival days, which suggests that socio-cultural events may influence ART outcomes.

In previous studies, the emphasis has been primarily on the impact of the endometrium and the embryo on assisted reproductive outcomes^23^. However, our study introduces an alternative perspective: external factors may exert a negative influence on the pregnancy outcomes of ART. In this study, we examined a multitude of social factors, including the educational level, occupational status, and residential location of patients, that could potentially influence the outcomes of ART. It has been shown that educational level is a significant factor in access to health care outcomes^24,25^. In our data, the population that experienced ET during the Spring Festival period had slightly higher education levels than those who underwent ET at other times. However, this difference was no longer present after PSM. A retrospective analysis conducted in China indicated that a mother’s educational attainment did not correlate with the probability of a successful live birth among patients undergoing either fresh or FET procedures^26^.

Our study also revealed that a higher percentage of employed patients opted for treatment during the Spring Festival compared to other periods. These findings indicate that, driven by their occupational status, working individuals prefer to utilize the extended holiday to undergo ART procedures. Moreover, an observational study reported that women who lived within 15 km of a fertility clinic were 21% more likely to undergo ART treatment than those who lived over 60 km away^27^. In our clinical center, the proportion of patients residing in Changsha undergoing IVF during the Spring Festival was greater than that during the non-festival periods. This suggests that a combination of geographical and holiday-related factors could potentially serve as confounding influences on women’s access to ART treatments.

To mitigate the impact of potential confounding bias as much as possible, we employed both PSM and multivariate logistic regression analysis to assess the impact of the “Spring Festival Effect” on pregnancy outcomes. We observed several interesting findings regarding the influence of socio-cultural factors on ART outcomes. In alignment with our observations, another fertility center in China has also reported that the live birth rate among patients who underwent ART during the Spring Festival period was statistically lower compared to those who underwent ET outside of this festive season^28^. In contrast to our results, a meta-analysis of prospective psychosocial studies demonstrated that emotional stress induced by fertility problems and life events does not compromise the chance of becoming pregnant^29^. This discrepancy may be attributed to the fact that the “festival effect” causes disturbances to both patients and doctors, rather than disturbing patients alone.

Furthermore, our analysis indicated that neither the National Day nor the Labour Day appeared to have a significant impact on the clinical outcomes of patients undergoing their first ET, with the exception of a reduced preterm birth rate observed during the National Day. These findings may be partially attributed to the relatively small sample size. Nevertheless, it also suggests that the Spring Festival, as the most significant traditional holiday in China, holds a more profound influence on daily life and behavior, including those of ART patients undergoing their first ET.

Several possible mechanisms may help explain why the Spring Festival could lead to unfavorable outcomes of ART. From the patients’ perspective, poor lifestyle habits during the festival (e.g., sleep deprivation, poor sleep quality, irregular diet, and binge eating) may negatively affect IVF/ICSI outcomes. A prospective study suggested that unhealthy sleep characteristics such as short nocturnal sleep, inappropriate sleep time, poor subjective sleep quality might impair occyte quantity, maturity, and fertilization, and reduce the chances of clinical pregnancy^30^. In addition, patients may find it difficult to strictly follow medication instructions under the holiday atmosphere. A population-based retrospective cohort study found that patients discharged during the December holiday period were less inclined to have timely outpatient follow-up and had a higher risk of readmission and death. This suggested that patients affected by the holiday period were more likely to deviate from medical orders, leading to adverse outcomes^31^.

From the clinician’s point of view, physicians’ attention might be distracted by festival events such as discussions on holiday-related topics, visits, and gift preparations, etc. An observational study comparing patients’ mortality after surgery on surgeons’ birthdays with that on other days showed higher postoperative mortality on surgeon’s birthday^32^. On the basis of these findings, it is possible that doctors may be distracted by life events unrelated to work, which may adversely affect patient prognosis.

### Strengths and limitations

The strengths of this study include its sufficient sample size and the use of PSM to minimize bias as much as possible. Good covariate balance was achieved in all baseline characteristics after PSM. We evaluated a new candidate factor influencing ART outcome from a novel perspective, which is often underestimated but has clinical guiding significance.

Despite these strengths, this study has several limitations. First, in view of the intrinsic property of this retrospective study, potential confounding factors cannot be excluded. After matching, the Non-Festival group was reduced from 10,751 to 3,354 cases, with a matching ratio of 31.2%, which indicates incomplete matching and may have introduced bias. However, the Festival group retained 98.4% of samples, indicating that nearly all samples in the Festival group were successfully matched. Hence, the results should be interpreted with caution. Second, while we identified an association between the Spring Festival effect and adverse pregnancy outcomes, we did not further explore the specific contributing factors. In the future, prospective questionnaire-based studies are warranted to investigate potential influences on ART outcomes during the Spring Festival.

## Conclusion

In conclusion, we demonstrated that sociocultural events, as exemplified by the Spring Festival, were associated with poor prognoses for patients undergoing IVF/ICSI. The clinical significance of this study underscores the need for clinicians to remain vigilant in patient care during major social events. Meanwhile, under the cheerful holiday atmosphere, patients should exercise stronger self-discipline to maintain healthy lifestyle habits that support favorable ART outcome.

## Electronic supplementary material

Below is the link to the electronic supplementary material.

Supplementary Material 1

Supplementary Material 2

## Data Availability

The datasets used and/or analyzed during the current study are available from the corresponding author on reasonable request.

## Abbreviations

AFC: Antral follicle count
AMH: Anti-Müllerian hormone
ART: Assisted reproductive technology
BMI: Body mass index
CI: Confidence interval
DOR: Diminished ovarian reserve
E2: Estradiol
ET: Embryo transfer
FBG: Fasting blood glucose
FET: Frozen-thawed embryo transfer
FINS: Fasting insulin
FSH: Follicle stimulating hormone
Gn: Gonadotrophin
GnRH: Gonadotrophin-releasing hormone
hCG: Human chorionic gonadotropin
HDL-C: High-density lipoprotein cholesterol
HOMA-IR: Homeostatic model assessment for insulin resistance
HRT: Hormone replacement therapy
ICSI: Intracytoplasmic sperm injection
IVF-ET: In vitro fertilization and embryo transfer
IQR: Interquartile range
LDL-C: Low-density lipoprotein cholesterol
LH: Luteinizing hormone
OR: Odds ratio
P4: Progesterone
PSM: Propensity score matching
RSA: Recurrent spontaneous abortion
SD: Standard deviation
SMD: Standardized mean differences
T: Testosterone
TC: Total cholesterol
TG: Triglycerides
TSH: Thyroid-stimulating hormone
