# Supplementary material for: Impact of spring festival on pregnancy outcomes in patients undergoing first embryo transfer: a retrospective cohort study
Source: Sci Rep. 2025 Jul 2;15:23339. doi: 10.1038/s41598-025-05029-6 (PMC12222965; doi:10.1038/s41598-025-05029-6)
Supplement: Supplementary file 1 — Supplementary Material 1 [file 41598_2025_5029_MOESM1_ESM.docx]

**Supplemental Material of “****Impact of Spring Festival on pregnancy outcomes in patients undergoing first embryo transfer: a retrospective cohort study”**

**Supplementary table 1.Baseline characteristics of the study population before and after PSM.**

|  | **1:1 Propensity-Matched Cohort^a^** | | | | **1:2 Propensity-Matched Cohort^b^** | | | | **1:3 Propensity-Matched Cohort^c^** | | | |
| --- | --- | --- | --- | --- | --- | --- | --- | --- | --- | --- | --- | --- |
| Parameter | Festival Group  （N=861） | Non-Festival Group (N=861) | P Value | SMD | Festival Group  （N=862） | Non-Festival Group  (N=1707) | P Value | SMD | Festival Group  （N=861） | Non-Festival Group  (N=2561) | P Value | SMD |
| Maternal age at Embryo Transfer (years; mean (SD)) | 30.63 ± 4.23 | 30.76 ± 4.11 | 0.521 | 0.031 | 30.64 ± 4.22 | 30.57 ± 4.19 | 0.669 | 0.018 | 30.63 ± 4.23 | 30.57 ± 4.12 | 0.688 | 0.016 |
| Maternal BMI (kg/m2; mean (SD)) | 21.80± 2.55 | 21.85± 2.24 | 0.704 | 0.018 | 21.80± 2.55 | 21.79± 2.23 | 0.876 | 0.007 | 21.80± 2.55 | 21.86± 2.25 | 0.592 | 0.022 |
| Education, n (%) |  |  | 0.700 | 0.041 |  |  | 0.325 | 0.063 |  |  | 0.376 | 0.055 |
| Primary school and below | 42 (4.88%) | 44 (5.11%) |  |  | 42 (4.87%) | 97 (5.68%) |  |  | 42 (4.88%) | 131 (5.12%) |  |  |
| Secondary and High Schools | 479 (55.63%) | 494 (57.38%) |  |  | 479 (55.57%) | 982 (57.53%) |  |  | 479 (55.63%) | 1487 (58.06%) |  |  |
| College and above | 340 (39.49%) | 323 (37.51%) |  |  | 341 (39.56%) | 628 (36.79%) |  |  | 340 (39.49%) | 943 (36.82%) |  |  |
| Family residence, n (%) |  |  | 0.116 | 0.100 |  |  | 0.348 | 0.060 |  |  | 0.111 | 0.082 |
| Changsha City | 89 (10.34%) | 68 (7.90%) |  |  | 89 (10.32%) | 149 (8.73%) |  |  | 89 (10.34%) | 218 (8.51%) |  |  |
| Outside Changsha and within Hunan Province | 515 (59.81%) | 508 (59.00%) |  |  | 516 (59.86%) | 1020 (59.75%) |  |  | 515 (59.81%) | 1501 (58.61%) |  |  |
| Outside Hunan Province | 257 (29.85%) | 285 (33.10%) |  |  | 257 (29.81%) | 538 (31.52%) |  |  | 257 (29.85%) | 842 (32.88%) |  |  |
| Occupational status, n (%) |  |  | 0.918 | 0.034 |  |  | 0.139 | 0.100 |  |  | 0.905 | 0.030 |
| Employed | 352 (40.88%) | 343 (39.84%) |  |  | 352 (40.84%) | 663 (38.84%) |  |  | 352 (40.88%) | 1030 (40.22%) |  |  |
| Self-employed | 100 (11.61%) | 108 (12.54%) |  |  | 99 (11.48%) | 250 (14.65%) |  |  | 100 (11.61%) | 322 (12.57%) |  |  |
| Freelance | 126 (14.63%) | 130 (15.10%) |  |  | 126 (14.62%) | 260 (15.23%) |  |  | 126 (14.63%) | 373 (14.56%) |  |  |
| Housewife | 283 (32.87%) | 280 (32.52%) |  |  | 285 (33.06%) | 534 (31.28%) |  |  | 283 (32.87%) | 836 (32.64%) |  |  |
| Occupational exposure, n (%) |  |  | 0.259 | 0.054 |  |  | 0.705 | 0.016 |  |  | 0.546 | 0.024 |
| ‘Unexposed’ occupation | 795 (92.33%) | 782 (90.82%) |  |  | 796 (92.34%) | 1569 (91.92%) |  |  | 795 (92.33%) | 2348 (91.68%) |  |  |
| ‘Exposed’ occupation | 66 (7.67%) | 79 (9.18%) |  |  | 66 (7.66%) | 138 (8.08%) |  |  | 66 (7.67%) | 213 (8.32%) |  |  |
| Duration of infertility (years; median (interquartile range)) | 3 (2,5) | 3 (2,5) | 0.254 | 0.052 | 3 (2,5) | 3 (2,5) | 0.820 | 0.008 | 3 (2,5) | 3 (2,5) | 0.924 | 0.012 |
| Type of infertility, n (%) |  |  | 0.699 | 0.019 |  |  | 0.823 | 0.009 |  |  | 0.901 | 0.005 |
| Primary infertility | 401 (46.57%) | 409 (47.50%) |  |  | 403 (46.75%) | 806 (47.22%) |  |  | 401 (46.57%) | 1199 (46.82%) |  |  |
| Secondary infertility | 460 (53.43%) | 452 (52.50%) |  |  | 459 (53.25%) | 901 (52.78%) |  |  | 460 (53.43%) | 1362 (53.18%) |  |  |
| Gravidity (n; median (interquartile range)) | 1 (0,2) | 1 (0,2) | 0.346 | 0.090 | 1 (0,2) | 1 (0,2) | 0.784 | 0.018 | 1 (0,2) | 1 (0,2) | 0.412 | 0.048 |
| Parity (n; median (interquartile range)) | 0 (0,0) | 0 (0,0) | 0.082 | 0.070 | 0 (0,0) | 0 (0,0) | 0.160 | 0.050 | 0 (0,0) | 0 (0,0) | 0.116 | 0.054 |
| Main indication for ART |  |  | 0.966 | 0.057 |  |  | 0.929 | 0.068 |  |  | 0.998 | 0.027 |
| Tubal factor | 560 (65.04%) | 556 (64.58%) |  |  | 559 (64.85%) | 1136 (66.55%) |  |  | 560 (65.04%) | 1676 (65.44%) |  |  |
| Ovulation disorders | 78 (9.06%) | 68 (7.90%) |  |  | 78 (9.05%) | 151 (8.85%) |  |  | 78 (9.06%) | 218 (8.51%) |  |  |
| Endometriosis | 45 (5.23%) | 48 (5.57%) |  |  | 47 (5.45%) | 92 (5.39%) |  |  | 45 (5.23%) | 129 (5.04%) |  |  |
| DOR | 32 (3.72%) | 32 (3.72%) |  |  | 32 (3.71%) | 57 (3.34%) |  |  | 32 (3.72%) | 90 (3.51%) |  |  |
| Male factor | 116 (13.47%) | 125 (14.52%) |  |  | 116 (13.46%) | 224 (13.12%) |  |  | 116 (13.47%) | 355 (13.87%) |  |  |
| Unexplained | 0 (0.00%) | 0 (0.00%) |  |  | 0 (0.00%) | 1 (0.06%) |  |  | 0 (0.00%) | 0 (0.00%) |  |  |
| RSA | 10 (1.16%) | 9 (1.05%) |  |  | 10 (1.16%) | 18 (1.05%) |  |  | 10 (1.16%) | 31 (1.21%) |  |  |
| Chromosomal abnormality | 20 (2.32%) | 23 (2.67%) |  |  | 20 (2.32%) | 28 (1.64%) |  |  | 20 (2.32%) | 62 (2.42%) |  |  |
| AMH, ng/ml; median (IQR) | 3.43 (2.02,5.58) | 3.47 (2.01,5.83) | 0.455 | 0.106 | 3.24 (1.90,5.26) | 3.40 (1.97,5.83) | 0.788 | 0.054 | 3.43 (2.02,5.58) | 3.64 (2.00,5.89) | 0.210 | 0.099 |
| Basal FSH, mIU/ml; median (IQR) | 6.30 (5.30,7.64) | 6.31 (5.30,7.62) | 0.611 | 0.050 | 6.31 (5.31,7.72) | 6.40 (5.26,7.60) | 0.496 | 0.022 | 6.30 (5.30,7.64) | 6.30 (5.30,7.60) | 0.779 | 0.031 |
| Basal LH, mIU/ml; median (IQR) | 4.81 (3.70,6.38) | 5.20 (3.75,7.00) | 0.123 | 0.101 | 4.73 (3.69,6.39) | 5.20 (3.70,7.19) | 0.425 | 0.065 | 4.81 (3.70,6.38) | 5.10 (3.70,6.90) | 0.132 | 0.095 |
| Basal estradiol  , pg/ml; median (IQR) | 34.50  (26.19,45.93) | 34.70  (25.93,45.46) | 0.932 | 0.016 | 35.46 (26.90,46.70) | 34.42 (25.87,45.79) | 0.638 | 0.021 | 34.50 (26.19,45.93) | 34.80  (26.28,44.69) | 0.814 | 0.005 |
| Testosterone  , ng/mL; median (IQR) | 0.25 (0.17,0.34) | 0.24 (0.16,0.33) | 0.291 | 0.010 | 0.26 (0.17,0.35) | 0.25 (0.18,0.35) | 0.968 | 0.017 | 0.25 (0.17,0.34) | 0.23 (0.17,0.33) | 0.257 | 0.004 |
| AFC, n (%) |  |  | 0.507 | 0.074 |  |  | 0.248 | 0.086 |  |  | 0.216 | 0.084 |
| 1-6 | 81 (9.41%） | 96 (11.15%) |  |  | 81 (9.40%） | 200 (11.72%) |  |  | 81 (9.41%） | 294 (11.48%) |  |  |
| 7-12 | 252 (29.27%) | 262 (30.43%) |  |  | 251 (29.12%) | 514 (30.11%) |  |  | 252 (29.27%) | 789 (30.81%) |  |  |
| 13-24 | 507 (58.89%) | 486 (56.45%) |  |  | 509 (59.05%) | 951 (55.71%) |  |  | 507 (58.89%) | 1419 (55.41%) |  |  |
| >24 | 21 (2.44%) | 17 (1.97%) |  |  | 21 (2.44%) | 42 (2.46%) |  |  | 21 (2.44%) | 59 (2.30%) |  |  |
| TSH, uIU/ml;  median (IQR) | 2.20 (1.50,2.99) | 2.21 (1.59,3.08) | 0.770 | 0.043 | 2.10 (1.49,3.04) | 2.18 (1.49,3.04) | 0.832 | 0.005 | 2.20 (1.50,2.99) | 2.20 (1.53,3.05) | 0.717 | 0.048 |
| TG (mmol/L;  median (IQR)) | 0.99 (0.76,1.46) | 1.05 (0.77,1.48) | 0.356 | 0.004 | 1.01 (0.78,1.52) | 1.02 (0.79,1.51) | 0.384 | 0.016 | 0.99 (0.76,1.46) | 1.05 (0.78,1.44) | 0.116 | 0.022 |
| TC (mmol/L;  median (IQR)) | 4.59 (4.10,5.19) | 4.65 (4.15,5.19) | 0.989 | 0.002 | 4.61 (4.12,5.11) | 4.59 (4.05,5.23) | 0.742 | 0.012 | 4.59 (4.10,5.19) | 4.66 (4.17,5.20) | 0.359 | 0.034 |
| LDL-C (mmol/L; median (IQR)) | 2.74 (2.32,3.18) | 2.73 (2.33,3.17) | 0.412 | 0.040 | 2.76 (2.36,3.17) | 2.70 (2.31,3.19) | 0.353 | 0.025 | 2.74 (2.32,3.18) | 2.76 (2.36,3.20) | 0.786 | 0.016 |
| HDL-C  (mmol/L; median (IQR)) | 1.40 (1.20,1.64) | 1.42 (1.24,1.64) | 0.091 | 0.099 | 1.34 (1.16,1.59) | 1.40 (1.19,1.61) | 0.443 | 0.053 | 1.40 (1.20,1.64) | 1.42 (1.22,1.64) | 0.147 | 0.080 |
| Dyslipidemia, (%, n) | 38.80% (247/637) | 37.30% (240/643) | 0.593 | 0.030 | 38.70% (247/639) | 39.40% (488/1239) | 0.758 | 0.015 | 38.80% (247/637) | 39.10% (735/1882) | 0.901 | 0.006 |
| FBG (mmol/L;  median (IQR)) | 5.24 (4.97,5.53) | 5.29 (5.03,5.57) | 0.193 | 0.077 | 5.23 (4.94,5.53) | 5.26 (4.98,5.55) | 0.633 | 0.028 | 5.24 (4.97,5.53) | 5.27 (5.01,5.55) | 0.462 | 0.046 |
| FINS (uU/mL; median (IQR)) | 8.87(6.42,13.03) | 9.52 (6.71,12.58) | 0.461 | 0.016 | 8.92 (6.39,13.33) | 9.25 (6.55,12.50) | 0.448 | 0.042 | 8.87 (6.42,13.03) | 9.47 (6.71,13.00) | 0.079 | 0.056 |
| HOMA-IR, (%, n) |  |  | 0.405 | 0.047 |  |  | 0.761 | 0.015 |  |  | 0.129 | 0.070 |
| <2.5 | 62.60% (395/631) | 60.30%  (380/630) |  |  | 62.80% (396/631) | 62.00% (773/1246) |  |  | 62.60%  (395/631) | 59.20%  (1115/1884) |  |  |
| ≥2.5 | 37.40% (236/631) | 39.70%  (250/630) |  |  | 37.20% (235/631) | 38.00% (473/1246) |  |  | 37.40%  (236/631) | 40.80%  (769/1884) |  |  |
| Type of cycle,  (%, n) |  |  | 0.960 | 0.002 |  |  | 0.513 | 0.027 |  |  | 0.956 | 0.002 |
| Cycle with fresh embryo transfer | 62.14% (535/861) | 62.25%  (536/861) |  |  | 62.06%  (535/862) | 63.39% (1082/1707) |  |  | 62.14%  (535/861) | 62.24%  (1594/2561) |  |  |
| Cycle with frozen-thawed embryo transfer | 37.86% (326/861) | 37.75%  (325/861) |  |  | 37.94%  (327/862) | 36.61% (625/1707) |  |  | 37.86%  (326/861) | 37.76%  (967/2561) |  |  |
| Fertilization method, n (%) |  |  | 0.705 | 0.040 |  |  | 0.552 | 0.046 |  |  | 0.772 | 0.029 |
| IVF | 627 (72.82%) | 614 (71.31%) |  |  | 628 (72.85%) | 1232 (72.17%) |  |  | 627 (72.82%) | 1839 (71.81%) |  |  |
| ICSI | 185 (21.49%) | 191 (22.18%) |  |  | 185 (21.46%) | 359 (21.03%) |  |  | 185 (21.49%) | 561 (21.91%) |  |  |
| IVF+ICSI | 49 (5.69%) | 56 (6.50%) |  |  | 49 (5.68%) | 116 (6.80%) |  |  | 49 (5.69%) | 161 (6.29%) |  |  |
| Endometrial thickness (mm; mean (SD)) | 10.47 ± 2.19 | 10.45 ± 2.14 | 0.798 | 0.012 | 10.47 ± 2.19 | 10.53 ± 2.15 | 0.507 | 0.028 | 10.47 ± 2.19 | 10.48 ± 2.17 | 0.896 | 0.005 |
| Endometrial type, n (%) |  |  | 0.828 | 0.030 |  |  | 0.402 | 0.057 |  |  | 0.349 | 0.057 |
| A | 319 (37.05%) | 331 (38.44%) |  |  | 319 (37.01%) | 678 (39.72%) |  |  | 319 (37.05%) | 1017 (39.71%) |  |  |
| B | 476 (55.28%) | 467 (54.24%) |  |  | 478 (55.45%) | 902 (52.84%) |  |  | 476 (55.28%) | 1345 (52.52%) |  |  |
| C | 66 (7.67%) | 63 (7.32%) |  |  | 65 (7.54%) | 127 (7.44%) |  |  | 66 (7.67%) | 199 (7.77%) |  |  |
| Stage of embryo development, n (%) |  |  | 0.625 | <0.001 |  |  | 0.858 | 0.026 |  |  | 0.352 | 0.001 |
| Cleavage stage embryo | 1300 (87.01%) | 1297 (86.41%) |  |  | 1300 (86.90%) | 2570 (87.09%) |  |  | 1300 (87.01%) | 3827 (86.06%) |  |  |
| Blastocyst stage embryos | 194 (12.99%) | 204 (13.59%) |  |  | 196 (13.10%) | 381 (12.91%) |  |  | 194 (12.99%) | 620 (13.94%) |  |  |
| No. of embryos transferred (n; mean (SD)) | 1.74±0.44 | 1.74±0.44 | 0.701 | 0.019 | 1.74±0.44 | 1.73±0.45 | 0.716 | 0.015 | 1.74±0.44 | 1.74±0.44 | 0.943 | 0.003 |
| Good-quality embryos transferred rate, (n, %) | 89.16% (1332/1494) | 89.41%  (1342/1501) | 0.825 | 0.020 | 88.97% (1331/1496) | 89.16% (2631/2951) | 0.851 | 0.025 | 89.16% (1332/1494) | 89.07%  (3961/4447) | 0.927 | 0.051 |
| Clinician’s title |  |  | 0.508 | 0.032 |  |  | 0.767 | 0.012 |  |  | 0.113 | 0.062 |
| Associate chief physician and above | 683 (79.33%) | 694 (80.60%) |  |  | 684 (79.35%) | 1363 (79.85%) |  |  | 683 (79.33%) | 2094 (81.76%) |  |  |
| Attending physician | 178 (20.67%) | 167 (19.40%) |  |  | 178 (20.65%) | 344 (20.15%) |  |  | 178 (20.67%) | 467 (18.24%) |  |  |
| Clinician’s gender |  |  | 0.751 | 0.015 |  |  | 0.192 | 0.054 |  |  | 0.168 | 0.054 |
| Female | 604 (70.15%) | 610 (70.85%) |  |  | 604 (70.07%) | 1238 (72.52%) |  |  | 604 (70.15%) | 1859 (72.59%) |  |  |
| Male | 257 (29.85%) | 251 (29.15%) |  |  | 258 (29.93%) | 469 (27.48%) |  |  | 257 (29.85%) | 702 (27.41%) |  |  |
| Abbreviations: PSM, Propensity Score Matching; SD, Standard Deviation; SMD, Standardized Mean Differences; BMI, Body Mass Index; ART, Assisted Reproductive Technology; DOR, Diminished Ovarian Reserve; RSA, Recurrent Spontaneous Abortion; AMH, anti-Mullerian Hormone; FSH, Follicle Stimulating Hormone; LH, Luteinizing Hormone; AFC, Antral Follicle Count; TSH, thyroid-stimulating hormone; TG, Triglyceride; TC: Total Cholesterol; LDL-C, Low-Density Lipoprotein Cholesterol; HDL-C, High-Density Lipoprotein Cholesterol; FBG, Fasting Blood Glucose; FINS, Fasting Insulin; HOMA-IR, Homeostatic Model Assessment for Insulin Resistance; IVF, In Vitro Fertilization; ICSI, Intracytoplasmic Sperm Injection; IQR, Interquartile Range  ^a^1:1 PSM was matched for maternal age at ET, maternal BMI, occupational status, duration of infertility, main indication for ART, type of cycle, stage of embryo development, number of embryos transferred and good-quality embryos.  ^b^1:2 PSM was matched for maternal age at ET, maternal BMI, duration of infertility, main indication for ART, type of cycle, endometrial type, stage of embryo development, number of embryos transferred, good-quality embryos and clinician’s title.  ^c^1:3 PSM was matched for maternal age at ET, maternal BMI, occupational status, duration of infertility, main indication for ART, type of cycle, stage of embryo development, number of embryos transferred and good-quality embryos. | | | | | | | | | | | | |

| **Supplementary table 2. Pregnancy outcomes of the study population before and after PSM** | | | | | | | | | |
| --- | --- | --- | --- | --- | --- | --- | --- | --- | --- |
|  | **1:1 Propensity-Matched Cohort^a^** | | | **1:2 Propensity-Matched Cohort^b^** | | | **1:3 Propensity-Matched Cohort^c^** | | |
| Variables | Festival Group  （N=861） | Non-Festival Group  (N=861) | P Value | Festival Group （N=862） | Non-Festival  Group  (N=1707) | P Value | Festival Group （N=861） | Non-Festival  Group  (N=2561) | P Value |
| Clinical pregnancy rate | 44.25% (381/861) | 50.17% (432/861) | 0.014 | 44.43% (383/862) | 49.09% (838/1707) | 0.026 | 44.25% (381/861) | 50.80% (1301/2561) | 0.001 |
| Positive β-hCG rate | 54.70% (471/861) | 58.95% (507/861) | 0.080 | 54.87% (473/862) | 59.75% (1020/1707) | 0.018 | 54.70% (471/861) | 60.80% (1557/2561) | 0.002 |
| Biochemical pregnancy rate | 8.94% (77/861) | 7.90% (68/861) | 0.435 | 8.93% (77/862) | 9.02% (154/1707) | 0.941 | 8.94% (77/861) | 8.47% (217/2561) | 0.670 |
| Implantation rate | 34.34% (513/1494） | 38.37% (576/1501） | 0.022 | 34.49%  (516/1496） | 38.56% (1138/2951） | 0.008 | 34.34%  (513/1494） | 39.89%  (1774/4447） | <0.001 |
| Missing data on transfer outcome | 0.70% (6/861) | 0.80% (7/861) | 0.781 | 0.81% (7/862) | 0.60% (10/1707) | 0.504 | 0.70% (6/861) | 0.98% (25/2561) | 0.454 |
| Ectopic pregnancy rate | 1.90% (16/861) | 0.80% (7/861) | 0.059 | 4.18% (16/383) | 3.46% (29/838) | 0.537 | 1.86% (16/861) | 1.56% (40/2561) | 0.553 |
| Miscarriage rate | 15.22% (58/381) | 12.73% (55/432) | 0.305 | 15.14% (58/383) | 12.05% (101/838) | 0.136 | 15.22% (58/381) | 14.22% (185/1301) | 0.624 |
| Early miscarriage rate | 12.07% (46/381) | 8.33% (36/432) | 0.077 | 12.01% (46/383) | 9.67% (81/838) | 0.213 | 12.07% (46/381) | 10.15% (132/1301) | 0.282 |
| Late miscarriage rate | 3.15% (12/381) | 4.40% (19/432) | 0.354 | 3.13% (12/383) | 2.39% (20/838) | 0.449 | 3.15% (12/381) | 4.07% (53/1301) | 0.410 |
| Ongoing pregnancy rate | 38.48% (329/855) | 45.55% (389/854) | 0.003 | 38.71% (331/855) | 44.02% (747/1697) | 0.010 | 38.48% (329/855) | 45.11% (1144/2536) | 0.001 |
| Preterm birth rate | 7.49% (64/855) | 8.67% (74/854) | 0.371 | 7.60% (65/855) | 9.13% (155/1697) | 0.193 | 7.49% (64/855) | 8.32% (211/2536) | 0.439 |
| Term birth rate | 29.59% (253/855) | 34.66% (296/854) | 0.025 | 29.71% (254/855) | 33.71% (572/1697) | 0.042 | 29.59% (253/855) | 34.66% (879/2536) | 0.007 |
| Live birth rate | 37.08% (317/855) | 43.33% (370/854) | 0.008 | 37.31% (319/855) | 42.66% (724/1697) | 0.009 | 37.08% (317/855) | 42.94% (1089/2536) | 0.003 |
| Abbreviations: PSM, Propensity Score Matching; hCG, human chorionic gonadotropin  ^a^1:1 PSM was matched for maternal age at ET, maternal BMI, occupational status, duration of infertility, main indication for ART, type of cycle, stage of embryo development, number of embryos transferred and good-quality embryos.  ^b^1:2 PSM was matched for maternal age at ET, maternal BMI, duration of infertility, main indication for ART, type of cycle, endometrial type, stage of embryo development, number of embryos transferred, good-quality embryos and clinician’s title.  ^c^1:3 PSM was matched for maternal age at ET, maternal BMI, occupational status, duration of infertility, main indication for ART, type of cycle, stage of embryo development, number of embryos transferred and good-quality embryos. | | | | | | | | | |

| **Supplementary table 3. Univariate and multivariate logistic regression analysis for factors associated with positive β-hCG** | | | | |
| --- | --- | --- | --- | --- |
| **Variable** | **Univariate Binary Logistic Regression** | | **Multivariate Logistic Regression ^a^** | |
|  | **Crude OR (95% CI)** | **P Value** | **Adjusted OR (95% CI)** | **P Value** |
| The period of embryo transfer, n (%) |  |  |  |  |
| Non-Festival Group | Reference |  | Reference |  |
| Festival Group | 0.732(0.637,0.842) | <0.001 | 0.729(0.631,0.841) | <0.001 |
| Maternal age at Embryo Transfer(years) | 0.942(0.933,0.950) | <0.001 | 0.954(0.944,0.965) | <0.001 |
| Maternal BMI (kg/m2; mean (SD)) | 0.993(0.978,1.009) | 0.415 |  |  |
| Education, n (%) |  | <0.001 |  | 0.031 |
| Primary school and below | Reference |  | Reference |  |
| Secondary and High Schools | 1.337(1.137,1.571) | <0.001 | 1.165(0.984,1.379) | 0.076 |
| College and above | 1.402(1.186,1.657) | <0.001 | 1.273(1.057,1.532) | 0.011 |
| Family residence, n (%) |  | 0.314 |  |  |
| Changsha City | Reference |  |  |  |
| Outside Changsha and within Hunan Province | 0.940(0.817,1.082) | 0.390 |  |  |
| Outside Hunan Province | 0.900(0.777,1.043) | 0.160 |  |  |
| Occupational status, n (%) |  | 0.001 |  | 0.031 |
| Employed | Reference |  | Reference |  |
| Self-employed | 0.846(0.754,0.948) | 0.004 | 0.908(0.797,1.034) | 0.144 |
| Freelance | 0.908(0.810,1.018) | 0.098 | 0.927(0.817,1.051) | 0.237 |
| Housewife | 1.056(0.965,1.155) | 0.236 | 1.062(0.954,1.181) | 0.273 |
| Occupational exposure, n (%) |  |  |  |  |
| ‘Unexposed’ occupation | Reference |  |  |  |
| ‘Exposed’ occupation | 0.977(0.848,1.126) | 0.751 |  |  |
| Duration of infertility (years) | 0.973(0.961,0.985) | <0.001 | 0.998(0.985,1.012) | 0.795 |
| Type of infertility, n (%) |  |  |  |  |
| Primary infertility | Reference |  | Reference |  |
| Secondary infertility | 0.825(0.765,0.889) | <0.001 | 1.072(0.984,1.168) | 0.110 |
| Gravidity (n; median (interquartile range)) | 0.913(0.887,0.940) | <0.001 |  |  |
| Parity (n; median (interquartile range)) | 0.806(0.746,0.870) | <0.001 |  |  |
| Main indication for ART, n (%) |  | <0.001 |  | 0.001 |
| Tubal factor | Reference |  | Reference |  |
| Ovulation disorders | 1.399(1.217,1.607) | <0.001 | 1.191(1.025,1.384) | 0.022 |
| Endometriosis | 0.967(0.805,1.163) | 0.725 | 0.994(0.82,1.205) | 0.952 |
| DOR | 0.623(0.511,0.760) | <0.001 | 0.852(0.69,1.053) | 0.138 |
| Male factor | 1.143(1.019,1.281) | 0.022 | 1.200(1.033,1.394) | 0.017 |
| RSA | 0.774(0.581,1.031) | 0.079 | 1.432(0.916,2.238) | 0.115 |
| Chromosomal abnormality | 1.110(0.727,1.695) | 0.628 | 0.858(0.618,1.192) | 0.362 |
| Unexplained infertility | 3.020(1.151,7.924) | 0.025 | 3.761(1.402,10.089) | 0.009 |
| AMH, ng/ml; median (IQR) | 1.055(1.041,1.069) | <0.001 |  |  |
| Basal FSH, mIU/ml; median (IQR) | 0.958(0.942,0.974) | <0.001 | 0.974(0.956,0.992) | 0.006 |
| Basal LH, mIU/ml; median (IQR) | 1.036(1.025,1.048) | <0.001 | 1.03(1.017,1.043) | <0.001 |
| Basal estradiol, pg/ml; median (IQR) | 0.998(0.996,1.000) | 0.053 |  |  |
| Testosterone, ng/mL; median (IQR) | 2.292(1.750,3.001) | <0.001 |  |  |
| AFC, n (%) |  | <0.001 |  |  |
| 1-6 | Reference |  |  |  |
| 7-12 | 1.641(1.445,1.863) | <0.001 |  |  |
| 13-24 | 2.078(1.842,2.343) | <0.001 |  |  |
| >24 | 2.118(1.593,2.815) | <0.001 |  |  |
| Thyroid-stimulating hormone, uIU/ml; median (IQR) | 0.998(0.978,1.019) | 0.870 |  |  |
| Serum Triglyceride (mmol/L; median (IQR)) | 0.987(0.938,1.038) | 0.602 |  |  |
| Total Cholesterol (mmol/L; median (IQR)) | 0.976(0.928,1.027) | 0.350 |  |  |
| Low-Density Lipoprotein Cholesterol (mmol/L; median (IQR)) | 0.955(0.897,1.017) | 0.155 |  |  |
| High-Density Lipoprotein Cholesterol (mmol/L; median (IQR)) | 1.071(0.952,1.206) | 0.253 |  |  |
| Whether complicated with dyslipidemia, % (n/N) |  |  |  |  |
| non-dyslipidemia | Reference |  |  |  |
| dyslipidemia | 1.025(0.936,1.122) | 0.591 |  |  |
| Fasting Blood Glucose (mmol/L; median (IQR)) | 0.887(0.810,0.970) | 0.009 |  |  |
| Fasting Insulin (uU/mL; median (IQR)) | 1.001(0.994,1.009) | 0.735 |  |  |
| HOMA-IR, % (n/N) |  |  |  |  |
| <2.5 | Reference |  |  |  |
| ≥2.5 | 1.001(0.915,1.095) | 0.990 |  |  |
| Type of cycle, n (%) |  |  |  |  |
| Cycle with fresh embryo transfer | Reference |  |  |  |
| Cycle with frozen-thawed embryo transfer | 0.952(0.876,1.034) | 0.242 |  |  |
| Fertilization method, n (%) |  | 0.099 |  | 0.007 |
| IVF | Reference |  | Reference |  |
| ICSI | 0.964(0.877,1.059) | 0.440 | 0.869(0.764,0.989) | 0.033 |
| IVF+ICSI | 0.855(0.738,0.990) | 0.036 | 0.819(0.704,0.952) | 0.009 |
| Endometrial thickness  (mm; mean (SD)) | 1.081(1.062,1.100) | <0.001 | 1.094(1.074,1.115) | <0.001 |
| Endometrial type, n (%) |  | 0.064 |  | 0.152 |
| A | Reference |  | Reference |  |
| B | 0.983(0.909,1.063) | 0.674 | 0.990(0.913,1.073) | 0.803 |
| C | 0.834(0.717,0.972) | 0.020 | 0.857(0.732,1.004) | 0.056 |
| Stage of embryo development, n (%) |  |  |  |  |
| Cleavage stage embryos | Reference |  | Reference |  |
| Blastocyst stage embryos | 1.272(1.131,1.431) | <0.001 | 2.032(1.76,2.347) | <0.001 |
| No. of embryos transferred (n; mean (SD)) | 1.740(1.590,1.905) | <0.001 | 2.114(1.903,2.347) | <0.001 |
| No. of good quality embryos transferred, % (n/N) |  | <0.001 |  |  |
| 0 | Reference |  |  |  |
| 1 | 0.899(0.762,1.060) | 0.205 |  |  |
| >=2 | 1.640(1.406,1.913) | <0.001 |  |  |
| Clinician’s title, n (%) |  |  |  |  |
| Associate chief physician and above | Reference |  |  |  |
| Attending physician | 0.928(0.843,1.022) | 0.127 |  |  |
| Clinician’s gender, n (%) |  |  |  |  |
| Female | Reference |  |  |  |
| Male | 0.927(0.852,1.008) | 0.075 |  |  |
| ^a^The model was adjusted for maternal age at embryo transfer, education, occupational status, duration of infertility, type of infertility, main indication for ART, basal FSH, basal LH, fertilization method, endometrial thickness, endometrial type, stage of embryo development and number of embryos transferred. | | | | |

| **Supplementary table 4. Univariate and multivariate logistic regression analysis for factors associated with clinical pregnancy** | | | | |
| --- | --- | --- | --- | --- |
| **Variable** | **Univariate Binary Logistic Regression** | | **Multivariate Logistic Regression ^a^** | |
|  | **Crude OR (95% CI)** | **P Value** | **Adjusted OR (95% CI)** | **P Value** |
| The period of embryo transfer, n (%) |  |  |  |  |
| Non-Festival Group | Reference |  | Reference |  |
| Festival Group | 0.722(0.629,0.830) | <0.001 | 0.717(0.622,0.827) | <0.001 |
| Maternal age at Embryo Transfer(years) | 0.947(0.939,0.956) | <0.001 | 0.958(0.948,0.968) | <0.001 |
| Maternal BMI (kg/m2; mean (SD)) | 0.995(0.980,1.011) | 0.563 |  |  |
| Education, n (%) |  | <0.001 |  | 0.001 |
| Primary school and below | Reference |  | Reference |  |
| Secondary and High Schools | 1.251(1.065,1.470) | 0.006 | 1.109(0.937,1.312) | 0.229 |
| College and above | 1.404(1.189,1.658) | <0.001 | 1.303(1.084,1.566) | 0.005 |
| Family residence, n (%) |  | 0.171 |  |  |
| Changsha City | Reference |  |  |  |
| Outside Changsha and within Hunan Province | 0.889(0.776,1.109) | 0.091 |  |  |
| Outside Hunan Province | 0.873(0.757,1.007) | 0.062 |  |  |
| Occupational status, n (%) |  | <0.001 |  | 0.011 |
| Employed | Reference |  | Reference |  |
| Self-employed | 0.816(0.730,0.914) | <0.001 | 0.904(0.796,1.027) | 0.120 |
| Freelance | 0.886(0.792,0.990) | 0.032 | 0.932(0.824,1.053) | 0.258 |
| Housewife | 1.028(0.943,1.122) | 0.529 | 1.076(0.971,1.193) | 0.163 |
| Occupational exposure, n (%) |  |  |  |  |
| ‘Unexposed’ occupation | Reference |  |  |  |
| ‘Exposed’ occupation | 1.002(0.873,1.151) | 0.972 |  |  |
| Duration of infertility (years) | 0.978(0.967,0.990) | <0.001 | 1.004(0.990,1.017) | 0.575 |
| Type of infertility, n (%) |  |  |  |  |
| Primary infertility | Reference |  | Reference |  |
| Secondary infertility | 0.830(0.771,0.892) | <0.001 | 1.077(0.991,1.170) | 0.082 |
| Gravidity (n; median (interquartile range)) | 0.912(0.886,0.938) | <0.001 |  |  |
| Parity (n; median (interquartile range)) | 0.803(0.744,0.867) | <0.001 |  |  |
| Main indication for ART, n (%) |  | <0.001 |  | 0.001 |
| Tubal factor | Reference |  | Reference |  |
| Ovulation disorders | 1.370(1.201,1.562) | <0.001 | 1.211(1.051,1.396) | 0.008 |
| Endometriosis | 1.056(0.882,1,265) | 0.553 | 1.048(0.868,1.265) | 0.625 |
| DOR | 0.587(0.479,0.721) | <0.001 | 0.788(0.635,0.977) | 0.030 |
| Male factor | 1.158(1.037,1.293) | 0.009 | 1.182(1.022,1.367) | 0.024 |
| RSA | 0.918(0.690,1.220) | 0.555 | 1.247(0.812,1.917) | 0.313 |
| Chromosomal abnormality | 0.956(0.637,1.437) | 0.830 | 1.013(0.731,1.402) | 0.940 |
| Unexplained infertility | 2.511(1.111,5.675) | 0.027 | 2.974(1.284,6.888) | 0.011 |
| AMH, ng/ml; median (IQR) | 1.045(1.032,1.058) | <0.001 |  |  |
| Basal FSH, mIU/ml; median (IQR) | 0.955(0.939,0.971) | <0.001 | 0.972(0.954,0.990) | 0.003 |
| Basal LH, mIU/ml; median (IQR) | 1.025(1.015,1.036) | <0.001 | 1.020(1.008,1.031) | 0.001 |
| Basal estradiol, pg/ml; median (IQR) | 0.998(0.996,0.999) | 0.013 |  |  |
| Testosterone, ng/mL; median (IQR) | 1.083(1.397,2.327) | <0.001 |  |  |
| AFC, n (%) |  | <0.001 |  |  |
| 1-6 | Reference |  |  |  |
| 7-12 | 1.612(1.417,1.834) | <0.001 |  |  |
| 13-24 | 2.011(1.780,2.271) | <0.001 |  |  |
| >24 | 2.158(1.639,2.842) | <0.001 |  |  |
| Thyroid-stimulating hormone, uIU/ml; median (IQR) | 1.005(0.985,1.026) | 0.603 |  |  |
| Serum Triglyceride (mmol/L; median (IQR)) | 0.975(0.928,1.025) | 0.318 |  |  |
| Total Cholesterol (mmol/L; median (IQR)) | 0.993(0.945,1.043) | 0.778 |  |  |
| Low-Density Lipoprotein Cholesterol (mmol/L; median (IQR)) | 0.969(0.911,1.030) | 0.313 |  |  |
| High-Density Lipoprotein Cholesterol (mmol/L; median (IQR)) | 1.089(0.972,1.221) | 0.142 |  |  |
| Whether complicated with dyslipidemia, % (n/N) |  |  |  |  |
| non-dyslipidemia | Reference |  |  |  |
| dyslipidemia | 1.062(0.973,1.159) | 0.179 |  |  |
| Fasting Blood Glucose (mmol/L; median (IQR)) | 0.896(0.821,0.978) | 0.014 |  |  |
| Fasting Insulin (uU/mL; median (IQR)) | 1.004(0.996,1.011) | 0.310 |  |  |
| HOMA-IR, % (n/N) |  |  |  |  |
| <2.5 | Reference |  |  |  |
| ≥2.5 | 1.028(0.943,1.121) | 0.532 |  |  |
| Type of cycle, n (%) |  |  |  |  |
| Cycle with fresh embryo transfer | Reference |  |  |  |
| Cycle with frozen-thawed embryo transfer | 0.967(0.892,1.049) | 0.421 |  |  |
| Fertilization method, n (%) |  | 0.080 |  | 0.013 |
| IVF | Reference |  | Reference |  |
| ICSI | 1.004(0.916,1.101) | 0.925 | 0.906(0.799,1.028) | 0.125 |
| IVF+ICSI | 0.850(0.736,0.981) | 0.027 | 0.815(0.702,0.945) | 0.007 |
| Endometrial thickness  (mm; mean (SD)) | 1.097(1.078,1.117) | <0.001 | 1.110(1.090,1.130) | <0.001 |
| Endometrial type, n (%) |  | 0.003 |  | 0.008 |
| A | Reference |  | Reference |  |
| B | 0.966(0.896,1.043) | 0.378 | 0.969(0.896,1.048) | 0.427 |
| C | 0.769(0.662,0.894) | 0.001 | 0.781(0.668,0.913) | 0.002 |
| Stage of embryo development, n (%) |  |  |  |  |
| Cleavage stage embryos | Reference |  | Reference |  |
| Blastocyst stage embryos | 1.251(1.118,1.400) | <0.001 | 1.973(1.720,2.263) | <0.001 |
| No. of embryos transferred (n; mean (SD)) | 1.702(1.554,1.864) | <0.001 | 2.087(1.878,2.319) | <0.001 |
| No. of good quality embryos transferred, % (n/N) |  | <0.001 |  |  |
| 0 | Reference |  |  |  |
| 1 | 0.920(0.779,1.076) | 0.326 |  |  |
| >=2 | 1.616(1.385,1.885） | <0.001 |  |  |
| Clinician’s title, n (%) |  |  |  |  |
| Associate chief physician and above | Reference |  |  |  |
| Attending physician | 0.917(0.835,1.008) | 0.072 |  |  |
| Clinician’s gender, n (%) |  |  |  |  |
| Female | Reference |  |  |  |
| Male | 0.961(0.885,1,043) | 0.337 |  |  |
| ^a^The model was adjusted for maternal age at embryo transfer, education, occupational status, duration of infertility, type of infertility, main indication for ART, basal FSH, basal LH, fertilization method, endometrial thickness, endometrial type, stage of embryo development and number of embryos transferred. | | | | |

| **Supplementary table 5. Univariate and multivariate logistic regression analysis for factors associated with ongoing pregnancy** | | | | |
| --- | --- | --- | --- | --- |
| **Variable** | **Univariate Binary Logistic Regression** | | **Multivariate Logistic Regression ^a^** | |
|  | **Crude OR (95% CI)** | **P Value** | **Adjusted OR (95% CI)** | **P Value** |
| The period of embryo transfer, n (%) |  |  |  |  |
| Non-Festival Group | Reference |  | Reference |  |
| Festival Group | 0.712(0.618,0.820) | <0.001 | 0.708(0.612,0.819) | <0.001 |
| Maternal age at Embryo Transfer(years) | 0.938(0.930,0.947) | <0.001 | 0.948(0.938,0.958) | <0.001 |
| Maternal BMI (kg/m2; mean (SD)) | 0.991(0.976,1.007) | 0.278 |  |  |
| Education, n (%) |  | 0.002 |  | 0.001 |
| Primary school and below | Reference |  | Reference |  |
| Secondary and High Schools | 1.225(1.040,1.442) | 0.015 | 1.053(0.888,1.249) | 0.553 |
| College and above | 1.377(1.164,1.630) | <0.001 | 1.252(1.039,1.508) | 0.018 |
| Family residence, n (%) |  | 0.160 |  |  |
| Changsha City | Reference |  |  |  |
| Outside Changsha and within Hunan Province | 0.897(0.783,1.027) | 0.117 |  |  |
| Outside Hunan Province | 0.870(0.755,1.003) | 0.056 |  |  |
| Occupational status, n (%) |  | <0.001 |  | 0.005 |
| Employed | Reference |  | Reference |  |
| Self-employed | 0.808(0.721,0.905) | <0.001 | 0.901(0.793,1.025) | 0.113 |
| Freelance | 0.893(0.798,0.998) | 0.047 | 0.939(0.830,1.063) | 0.322 |
| Housewife | 1.045(0.958,1.140) | 0.318 | 1.094(0.986,1.213) | 0.089 |
| Occupational exposure, n (%) |  |  |  |  |
| ‘Unexposed’ occupation | Reference |  |  |  |
| ‘Exposed’ occupation | 1.006(0.876,1.156) | 0.929 |  |  |
| Duration of infertility (years) | 0.972(0.960,0.984) | <0.001 | 1.001(0.988,1.015) | 0.876 |
| Type of infertility, n (%) |  |  |  |  |
| Primary infertility | Reference |  | Reference |  |
| Secondary infertility | 0.806(0.750,0.868) | <0.001 | 1.073(0.988,1.167) | 0.095 |
| Gravidity (n; median (interquartile range)) | 0.901(0.875,0.927) | <0.001 |  |  |
| Parity (n; median (interquartile range)) | 0.759(0.701,0.821) | <0.001 |  |  |
| Main indication for ART, n (%) |  | <0.001 |  | <0.001 |
| Tubal factor | Reference |  | Reference |  |
| Ovulation disorders | 1.364(1.198,1.553) | <0.001 | 1.203(1.046,1.385) | 0.010 |
| Endometriosis | 1.070(0.894,1.282) | 0.46 | 1.060(0.878,1.280) | 0.542 |
| DOR | 0.531(0.428,0.658) | <0.001 | 0.720(0.574,0.902) | 0.004 |
| Male factor | 1.212(1.086,1.352) | 0.001 | 1.234(1.067,1.428) | 0.005 |
| RSA | 0.897(0.594,1.353) | 0.603 | 1.214(0.786,1.875) | 0.383 |
| Chromosomal abnormality | 0.924(0.693,1.232) | 0.591 | 1.043(0.751,1.448) | 0.802 |
| Unexplained infertility | 2.300(1.068,4.952) | 0.033 | 2.676(1.212,5.908) | 0.015 |
| AMH, ng/ml; median (IQR) | 1.044(1.031,1.056) | <0.001 |  |  |
| Basal FSH, mIU/ml; median (IQR) | 0.951(0.935,0.967) | <0.001 | 0.973(0.954,0.991) | 0.004 |
| Basal LH, mIU/ml; median (IQR) | 1.022(1.012,1.033) | <0.001 | 1.015(1.004,1.027) | 0.009 |
| Basal estradiol, pg/ml; median (IQR) | 0.998(0.996,0.999) | 0.009 |  |  |
| Testosterone, ng/mL; median (IQR) | 1.873(1.453,2.414) | <0.001 |  |  |
| AFC, n (%) |  | <0.001 |  |  |
| 1-6 | Reference |  |  |  |
| 7-12 | 1.752(1.532,2.004) | <0.001 |  |  |
| 13-24 | 2.184(1.924,2.480) | <0.001 |  |  |
| >24 | 2.697(2.045,3.557) | <0.001 |  |  |
| Thyroid-stimulating hormone, uIU/ml; median (IQR) | 1.005(0.985,1.025) | 0.635 |  |  |
| Serum Triglyceride (mmol/L; median (IQR)) | 0.965(0.918,1.014) | 0.160 |  |  |
| Total Cholesterol (mmol/L; median (IQR)) | 0.973(0.926,1.022) | 0.271 |  |  |
| Low-Density Lipoprotein Cholesterol (mmol/L; median (IQR)) | 0.970(0.913,1.032) | 0.338 |  |  |
| High-Density Lipoprotein Cholesterol (mmol/L; median (IQR)) | 0.977(0.872,1.095) | 0.693 |  |  |
| Whether complicated with dyslipidemia, % (n/N) |  |  |  |  |
| non-dyslipidemia | Reference |  |  |  |
| dyslipidemia | 1.028(0.942,1.112) | 0.539 |  |  |
| Fasting Blood Glucose (mmol/L; median (IQR)) | 0.902(0.826,0.984) | 0.021 |  |  |
| Fasting Insulin (uU/mL; median (IQR)) | 1.006(0.999,1.014) | 0.106 |  |  |
| HOMA-IR, % (n/N) |  |  |  |  |
| <2.5 | Reference |  |  |  |
| ≥2.5 | 1.060(0.971,1.156) | 0.191 |  |  |
| Type of cycle, n (%) |  |  |  |  |
| Cycle with fresh embryo transfer | Reference |  |  |  |
| Cycle with frozen-thawed embryo transfer | 0.922(0.850,1.000) | 0.050 |  |  |
| Fertilization method, n (%) |  | 0.164 |  | 0.03 |
| IVF | Reference |  | Reference |  |
| ICSI | 1.025(0.935,1.124) | 0.595 | 0.899(0.792,1.021) | 0.100 |
| IVF+ICSI | 0.879(0.760,1.017) | 0.083 | 0.841(0.724,0.977) | 0.024 |
| Endometrial thickness (mm; mean (SD)) | 1.101(1.082,1.120) | <0.001 | 1.112(1.092,1.133) | <0.001 |
| Endometrial type, n (%) |  | 0.004 |  | 0.019 |
| A | Reference |  | Reference |  |
| B | 0.951(0.881,1.026) | 0.191 | 0.955(0.883,1.032) | 0.246 |
| C | 0.774(0.664,0.901) | 0.001 | 0.798(0.682,0.935) | 0.005 |
| Stage of embryo development, n (%) |  |  |  |  |
| Cleavage stage embryos | Reference |  | Reference |  |
| Blastocyst stage embryos | 1.210(1.082,1.352) | <0.001 | 1.869(1.632,2.142) | <0.001 |
| No. of embryos transferred (n; mean (SD)) | 1.679(1.531,1.842) | <0.001 | 2.012(1.807,2.241) | <0.001 |
| No. of good quality embryos transferred, % (n/N) |  | <0.001 |  |  |
| 0 |  |  |  |  |
| 1 | 1.007(0.848,1.195) | 0.938 |  |  |
| >=2 | 1.727(1.475,2.023) | <0.001 |  |  |
| Clinician’s title, n (%) |  |  |  |  |
| Associate chief physician and above | Reference |  |  |  |
| Attending physician | 0.965(0.879,1.061) | 0.462 |  |  |
| Clinician’s gender, n (%) |  |  |  |  |
| Female | Reference |  |  |  |
| Male | 0.989(0.911,1.073) | 0.782 |  |  |
| ^a^The model was adjusted for maternal age at embryo transfer, education, occupational status, duration of infertility, type of infertility, main indication for ART, basal FSH, basal LH, fertilization method, endometrial thickness, endometrial type, stage of embryo development and number of embryos transferred. | | | | |

| **Supplementary table 6. Univariate and multivariate logistic regression analysis for factors associated with live birth** | | | | |
| --- | --- | --- | --- | --- |
| **Variable** | **Univariate Binary Logistic Regression** | | **Multivariate Logistic Regression ^a^** | |
|  | **Crude OR (95% CI)** | **P Value** | **Adjusted OR (95% CI)** | **P Value** |
| The period of embryo transfer, n (%) |  |  |  |  |
| Non-Festival Group | Reference |  | Reference |  |
| Festival Group | 0.736(0.638,0.849) | <0.001 | 0.731(0.631,0.847) | <0.001 |
| Maternal age at Embryo Transfer(years) | 0.938(0.930,0.947) | <0.001 | 0.947(0.937,0.957) | <0.001 |
| Maternal BMI (kg/m2; mean (SD)) | 0.986(0.971,1.001) | 0.072 |  |  |
| Education, n (%) |  | <0.001 |  | 0.002 |
| Primary school and below | Reference |  | Reference |  |
| Secondary and High Schools | 1.190(1.009,1.405) | 0.039 | 1.019(0.857,1.211) | 0.833 |
| College and above | 1.342(1.132,1.591) | 0.001 | 1.210(1.002,1.461) | 0.048 |
| Family residence, n (%) |  | 0.193 |  |  |
| Changsha City | Reference |  |  |  |
| Outside Changsha and within Hunan Province | 0.915(0.798,1.048) | 0.199 |  |  |
| Outside Hunan Province | 0.878(0.761,1.014) | 0.076 |  |  |
| Occupational status, n (%) |  | <0.001 |  | <0.001 |
| Employed | Reference |  | Reference |  |
| Self-employed | 0.779(0.694,0.874) | <0.001 | 0.864(0.759,0.984) | 0.028 |
| Freelance | 0.881(0.787,0.986) | 0.027 | 0.923(0.815,1.046) | 0.210 |
| Housewife | 1.060(0.971,1.157) | 0.192 | 1.107(0.997,1.228) | 0.056 |
| Occupational exposure, n (%) |  |  |  |  |
| ‘Unexposed’ occupation | Reference |  |  |  |
| ‘Exposed’ occupation | 1.037(0.902,1.192) | 0.613 |  |  |
| Duration of infertility (years) | 0.971(0.959,0.983) | <0.001 | 1.000(0.987,1.014) | 0.957 |
| Type of infertility, n (%) |  |  |  |  |
| Primary infertility | Reference |  |  |  |
| Secondary infertility | 0.818(0.759,0.880) | <0.001 | 1.086(0.998,1.181) | 0.055 |
| Gravidity (n; median (interquartile range)) | 0.903(0.877,0.930) | <0.001 |  |  |
| Parity (n; median (interquartile range)) | 0.750(0.693,0.812) | <0.001 |  |  |
| Main indication for ART, n (%) |  | <0.001 |  | <0.001 |
| Tubal factor | Reference |  | Reference |  |
| Ovulation disorders | 1.273(1.118,1.450) | <0.001 | 1.128(0.980,1.298) | 0.093 |
| Endometriosis | 1.077(.0898,1.290) | 0.424 | 1.063(0.880,1.284) | 0.527 |
| DOR | 0.506(0.406,0.630) | <0.001 | 0.680(0.540,0.856) | 0.001 |
| Male factor | 1.201(1.076,1.341) | 0.001 | 1.232(1.065,1.427) | 0.005 |
| RSA | 0.839(0.554,1.272) | 0.409 | 1.134(0.731,1.760) | 0.575 |
| Chromosomal abnormality | 0.915(0.685,1.223) | 0.55 | 1.029(0.740,1.432) | 0.864 |
| Unexplained infertility | 2.457(1.141,5.290) | 0.022 | 2.844(1.289,6.275) | 0.010 |
| AMH, ng/ml; median (IQR) | 1.041(1.029,1.054) | <0.001 |  |  |
| Basal FSH, mIU/ml; median (IQR) | 0.953(0.936,0.969) | <0.001 | 0.975(0.957,0.994) | 0.010 |
| Basal LH, mIU/ml; median (IQR) | 1.019(1.009,1.030) | <0.001 | 1.013(1.002,1.025) | 0.025 |
| Basal estradiol, pg/ml; median (IQR) | 0.998(0.996,0.999) | 0.015 |  |  |
| Testosterone, ng/mL; median (IQR) | 1.695(1.314,2.186) | <0.001 |  |  |
| AFC, n (%) |  | <0.001 |  |  |
| 1-6 | Reference |  |  |  |
| 7-12 | 1.773(1.546,2.032) | <0.001 |  |  |
| 13-24 | 2.182(1.917,2.484) | <0.001 |  |  |
| >24 | 2.737(2.074,3.611) | <0.001 |  |  |
| Thyroid-stimulating hormone, uIU/ml; median (IQR) | 1.008(0.988,1.029) | 0.424 |  |  |
| Serum Triglyceride (mmol/L; median (IQR)) | 0.941(0.893,0.991) | 0.021 |  |  |
| Total Cholesterol (mmol/L; median (IQR)) | 0.958(0.912,1.007) | 0.092 |  |  |
| Low-Density Lipoprotein Cholesterol (mmol/L; median (IQR)) | 0.953(0.895,1.013) | 0.124 |  |  |
| High-Density Lipoprotein Cholesterol (mmol/L; median (IQR)) | 0.989(0.881,1.109) | 0.846 |  |  |
| Whether complicated with dyslipidemia, % (n/N) |  |  |  |  |
| non-dyslipidemia | Reference |  |  |  |
| dyslipidemia | 0.982(0.899,1.073) | 0.690 |  |  |
| Fasting Blood Glucose (mmol/L; median (IQR)) | 0.889(0.814,0.971) | 0.009 |  |  |
| Fasting Insulin (uU/mL; median (IQR)) | 1.002(0.995,1.009) | 0.608 |  |  |
| HOMA-IR, % (n/N) |  |  |  |  |
| <2.5 | Reference |  |  |  |
| ≥2.5 | 1.021(0.936,1.115) | 0.638 |  |  |
| Type of cycle, n (%) |  |  |  |  |
| Cycle with fresh embryo transfer | Reference |  |  |  |
| Cycle with frozen-thawed embryo transfer | 0.921(0.849,1.000) | 0.049 |  |  |
| Fertilization method, n (%) |  | 0.125 |  | 0.017 |
| IVF | Reference |  | Reference |  |
| ICSI | 1.017(0.927,1.116) | 0.713 | 0.890(0.783,1.011) | 0.074 |
| IVF+ICSI | 0.865(0.746,1.001) | 0.053 | 0.829(0.712,0.964) | 0.015 |
| Endometrial thickness (mm; mean (SD)) | 1.105(1.086,1.124) | <0.001 | 1.116(1.095,1.136) | <0.001 |
| Endometrial type, n (%) |  | 0.002 |  | 0.013 |
| A | Reference |  | Reference |  |
| B | 0.948(0.878,1.023) | 0.168 | 0.952(0.879,1.030) | 0.218 |
| C | 0.762(0.653,0.889) | 0.001 | 0.788(0.672,0.925) | 0.004 |
| Stage of embryo development, n (%) |  |  |  |  |
| Cleavage stage embryos | Reference |  | Reference |  |
| Blastocyst stage embryos | 1.203(1.076,1.345) | <0.001 | 1.847(1.612,2.116) | <0.001 |
| No. of embryos transferred (n; mean (SD)) | 1.642(1.496,1.803) | <0.001 | 1.944(1.744,2.166) | <0.001 |
| No. of good quality embryos transferred, % (n/N) |  | <0.001 |  |  |
| 0 | Reference |  |  |  |
| 1 | 1.014(0.853,1.206) | 0.871 |  |  |
| >=2 | 1.694(1.444,1.987) | <0.001 |  |  |
| Clinician’s title, n (%) |  |  |  |  |
| Associate chief physician and above | Reference |  |  |  |
| Attending physician | 0.978(0.889,1.075) | 0.638 |  |  |
| Clinician’s gender, n (%) |  |  |  |  |
| Female | Reference |  |  |  |
| Male | 0.983(0.905,1.068) | 0.693 |  |  |
| ^a^The model was adjusted for maternal age at embryo transfer, education, occupational status, duration of infertility, type of infertility, main indication for ART, basal FSH, basal LH, fertilization method, endometrial thickness, endometrial type, stage of embryo development and number of embryos transferred. | | | | |

| **Supplementary table 7. Baseline characteristics of the study population who underwent embryo transfer on National Day or Labour Day.** | | | | | | |
| --- | --- | --- | --- | --- | --- | --- |
| **Characteristics** | **National Day Group**  **(N=196)** | **Non-National Day Group**  **(N=11429)** | **P Value** | **Labour Day Group**  **(N=108)** | **Non-Labour Day Group**  **(N=11517)** | **P Value** |
| Maternal age at Embryo Transfer (years; mean (SD)) | 30.40 ± 3.95 | 30.44 ± 4.18 | 0.901 | 30.44 ± 4.44 | 30.44 ± 4.17 | 0.800 |
| Maternal BMI (kg/m2; mean (SD)) | 22.19 ± 2.46 | 22.09 ± 2.36 | 0.657 | 22.27± 2.25 | 22.09 ± 2.36 | 0.343 |
| Education, n (%) |  |  | 0.749 |  |  | 0.695 |
| Primary school and below | 11(5.60%) | 642(5.60%) |  | 8(7.40%) | 645(5.60%) |  |
| Secondary and High Schools | 113(57.70%) | 6881(60.20%) |  | 65(60.20%) | 6929(60.20%) |  |
| College and above | 72(36.70%) | 3906(34.20%) |  | 35(32.40%) | 3943(34.20%) |  |
| Family residence, n (%) |  |  | 0.637 |  |  | 0.565 |
| Changsha City | 16(8.20%) | 936(8.20%) |  | 6(5.60%) | 946(8.20%) |  |
| Outside Changsha and within Hunan Province | 110(56.10%) | 6771(59.20%) |  | 64(59.30%) | 6817(59.20%) |  |
| Outside Hunan Province | 70(35.70%) | 3722(32.60%) |  | 38(35.20%) | 3754(32.60%) |  |
| Occupational status, n (%) |  |  | 0.691 |  |  | 0.421 |
| Employed | 76(38.80%) | 4188(36.60%) |  | 37(34.30%) | 4227(36.70%) |  |
| Self-employed | 23(11.70%) | 1678(14.70%) |  | 17(15.70%) | 1684(14.60%) |  |
| Freelance | 29(14.80%) | 1723(15.10%) |  | 22(20.40%) | 1730(15.00%) |  |
| Housewife | 68(34.70%) | 3840(33.60%) |  | 32(29.60%) | 3876(33.70%) |  |
| Occupational exposure, n (%) |  |  | 0.523 |  |  | 0.736 |
| ‘Unexposed’ occupation | 179(91.30%) | 10576(92.50%) |  | 99(91.70%) | 10656(92.52%) |  |
| ‘Exposed’ occupation | 17(8.70%) | 853(7.50%) |  | 9(8.30%) | 861(7.48%) |  |
| Duration of infertility (years; median (interquartile range)) | 3(2,5) | 3(2,5) | 0.086 | 3(2,5) | 3(2,5) | 0.541 |
| Type of infertility, n(%) |  |  | 0.107 |  |  | 0.077 |
| Primary infertility | 104(53.10%) | 5401(47.30%) |  | 42(38.90%) | 5463(47.40%) |  |
| Secondary infertility | 92(46.90%) | 6028(52.70%) |  | 66(61.10%) | 6054(52.60%) |  |
| Gravidity (n; median (interquartile range)) | 0(0,1) | 1(0,2) | 0.082 | 1(0,2) | 1(0,2) | 0.043 |
| Parity (n; median (interquartile range)) | 0(0,0) | 0(0,0) | 0.437 | 0(0,0) | 0(0,0) | 0.755 |
| Main indication for ART, n (%) |  |  | 0.224 |  |  | 0.496 |
| Tubal factor | 131(66.80%) | 7689(67.30%) |  | 80(74.10%) | 7740(67.20%) |  |
| Ovulation disorders | 22(11.20%) | 1019(8.90%) |  | 6(5.60%) | 1035(9.00%) |  |
| Endometriosis | 9(4.60%) | 496(4.30%) |  | 4(3.70%) | 501(4.40%) |  |
| DOR | 10(5.10%) | 400(3.50%) |  | 6(5.60%) | 404(3.50%) |  |
| Male factor | 18(9.20%) | 1514(13.20%) |  | 11(10.20%) | 1521(13.20%) |  |
| RSA | 0(0.00%) | 94(0.80%) |  | 1(0.90%) | 93(0.80%) |  |
| Chromosomal abnormality | 6(3.10%) | 188(1.60%) |  | 0(0.00%) | 194(1.70%) |  |
| Unexplained infertility | 0(0.00%) | 29(0.30%) |  | 0(0.00%) | 29(0.30%) |  |
| AMH, ng/ml; median (IQR) | 3.38(2.18,5.76) | 3.53(2.00,5.79) | 0.962 | 2.75(1.69,4.44) | 3.54(2.01,5.80) | 0.004 |
| Basal FSH, mIU/ml; median (IQR) | 6.45(5.36,7.50) | 6.30(5.29,7.50) | 0.489 | 6.40(5.17,7.29) | 6.30(5.30,7.50) | 0.682 |
| Basal LH, mIU/ml; median (IQR) | 5.10(3.67,7.28) | 4.90(3.54,6.60) | 0.132 | 5.10(3.40,6.92) | 4.90(3.54,6.60) | 0.580 |
| Basal estradiol, pg/ml; median (IQR) | 34.30(24.00,44.90) | 34.00(25.11,44.47) | 0.885 | 33.05(26.73,42.84) | 34.00(25.06,44.50) | 0.890 |
| Testosterone, ng/mL; median (IQR) | 0.22(0.14,0.31) | 0.24(0.16,0.33) | 0.066 | 0.25(0.17,0.35) | 0.24(0.16,0.33) | 0.308 |
| AFC, n (%) |  |  | 0.098 |  |  | 0.508 |
| 1-6 | 29(14.80%） | 1274(11.10%) |  | 15(13.90%） | 1288(11.20%) |  |
| 7-12 | 47(24.00%) | 3596(31.50%) |  | 29(26.90%) | 3614(31.40%) |  |
| 13-24 | 115(58.70%) | 6316(55.30%) |  | 63(58.30%) | 6368(55.30%) |  |
| >24 | 5(2.60%) | 243(2.10%) |  | 1(0.90%) | 247(2.10%) |  |
| Thyroid-stimulating hormone, uIU/ml; median (IQR) | 2.26(1.66,2.97) | 2.17(1.51,3.06) | 0.439 | 2.27(1.65,3.43) | 2.17(1.51,3.06) | 0.125 |
| Serum Triglyceride (mmol/L; median (IQR)) | 1.04(0.77,1.42) | 1.05(0.78,1.48) | 0.694 | 1.06(0.81,1.38) | 1.05(0.78,1.48) | 0.997 |
| Total Cholesterol (mmol/L;  median (IQR)) | 4.51(3.97,5.25) | 4.62(4.11,5.18） | 0.651 | 4.53(4.04,5.10) | 4.62(4.11,5.19） | 0.644 |
| Low-Density Lipoprotein Cholesterol (mmol/L; median (IQR)) | 2.70(2.23,3.20) | 2.72(2.29,3.18) | 0.633 | 2.75(2.40,3.21) | 2.72(2.29,3.18) | 0.835 |
| High-Density Lipoprotein Cholesterol (mmol/L; median (IQR)) | 1.39(1.19,1.59) | 1.41(1.20,1.64) | 0.384 | 1.38(1.18,1.62) | 1.41(1.20,1.64) | 0.335 |
| Dyslipidemia, % (n/N) | 41.30%(62/150) | 39.30%(3267/831) | 0.615 | 34.50% (29/84) | 39.40% (3300/8377) | 0.363 |
| Fasting Blood Glucose (mmol/L; median (IQR)) | 5.21(4.98,5.53) | 5.29(5.03,5.56) | 0.037 | 5.27(4.99,5.49) | 5.29(5.03,5.56) | 0.258 |
| Fasting Insulin (uU/mL; median (IQR)) | 9.79(6.78,12.88) | 9.53(6.90,13.17) | 0.746 | 9.15(6.75,12.83) | 9.53(6.90,13.16) | 0.724 |
| HOMA-IR, % (n/N) |  |  | 0.221 |  |  | 0.692 |
| <2.5 | 54.20% (83/153) | 59.20% (4904/8289) |  | 61.20% (52/85) | 59.10% (4588/8357) |  |
| ≥2.5 | 45.80% (70/153) | 40.80% (3385/8289) |  | 38.80% (33/85) | 41.20% (3218/8357) |  |
| Type of cycle, n (%) |  |  | 0.307 |  |  | 0.023 |
| Cycle with fresh embryo transfer | 68.36% (134/196) | 71.7% (8193/11429) |  | 81.48% (88/108) | 71.50%(8239/11517) |  |
| Cycle with frozen-thawed embryo transfer | 31.63% (62/196) | 28.3% (2965/11429) |  | 18.52% (20/108) | 28.50% (3278/11517) |  |
| Fertilization method, n (%) |  |  | 0.689 |  |  | 0.287 |
| IVF | 138(70.41%) | 8340(72.97%) |  | 86(79.60%) | 8392(72.90%) |  |
| ICSI | 42(21.43%) | 2294(20.07%) |  | 16(14.80%) | 2320(20.10%) |  |
| IVF+ICSI | 16(8.16%) | 795(6.96%) |  | 6(5.60%) | 805(7.00%) |  |
| Endometrial thickness (mm; mean (SD)) | 10.57 ± 2.26 | 10.58 ± 2.14 | 0.987 | 10.79 ± 2.47 | 10.58 ± 2.14 | 0.556 |
| Endometrial type, n (%) |  |  | 0.700 |  |  | 0.238 |
| A | 76(38.80%) | 4774(41.80%) |  | 46(42.60%) | 4804(41.70%) |  |
| B | 106(54.10%) | 5873(51.40%) |  | 59(54.60%) | 5920(51.40%) |  |
| C | 14(7.10%) | 782(6.80%) |  | 3(2.80%) | 793(6.90%) |  |
| Stage of embryo development, n (%) |  |  | 0.349 |  |  | 0.360 |
| Cleavage stage embryos | 168(85.70%) | 10048(87.90%) |  | 98(90.70%) | 10118(87.90%) |  |
| Blastocyst stage embryos | 28(14.30%) | 1381(12.10%) |  | 10(9.30%) | 1399(12.10%) |  |
| No. of embryos transferred (n; mean (SD)) | 1.78 ± 0.41 | 1.79 ± 0.41 | 0.690 | 1.82 ± 0.38 | 1.79 ± 0.41 | 0.411 |
| Good-quality embryos transferred rate, % (n/N) | 92.55%  (323/349) | 91.85%  (18815/20484) | 0.636 | 86.29%  (170/197) | 91.90%  (18968/20636) | 0.004 |
| Clinician’s title, n (%) |  |  | 0.024 |  |  | 0.784 |
| Associate chief physician and above | 172(87.80%) | 9311(81.50%) |  | 87(80.60%) | 9396(81.60%) |  |
| Attending physician | 24(12.20%) | 2118(18.50%) |  | 21(19.40%) | 2121(18.40%) |  |
| Clinician’s gender, n (%) |  |  | 0.704 |  |  | 0.007 |
| Female | 145(74.00%) | 8316(72.80%) |  | 91(84.30%) | 8370(72.70%) |  |
| Male | 51(26.00%) | 3113(27.20%) |  | 17(15.70%) | 3147(27.30%) |  |
| Abbreviations: SD, Standard Deviation; BMI, Body Mass Index; ART, Assisted Reproductive Technology; DOR, Diminished Ovarian Reserve; RSA, Recurrent Spontaneous Abortion; AMH, anti-Mullerian Hormone; FSH, Follicle Stimulating Hormone; LH, Luteinizing Hormone; AFC, Antral Follicle Count; HOMA-IR, Homeostatic Model Assessment for Insulin Resistance; IVF, In Vitro Fertilization; ICSI, Intracytoplasmic Sperm Injection; IQR, Interquartile Range | | | | | | |

| **Supplementary table 8.Pregnancy outcomes of the study population who underwent embryo transfer on National Day or Labour Day.** | | | | | | |
| --- | --- | --- | --- | --- | --- | --- |
| **Variables** | **National Day Group**  **(N=196)** | **Non-National Day Group**  **(N=11429)** | **P Value** | **Labour Day Group**  **(N=108)** | **Non-Labour**  **Day Group (N=11517)** | **P Value** |
| **Primary outcome** | | | | | | |
| Clinical pregnancy rate | 52.55% (103/196) | 51.89% (5930/11429) | 0.853 | 52.78% (57/108) | 51.89% (5976/11517) | 0.854 |
| Live birth rate | 43.08% (84/195) | 44.27% (5014/11325) | 0.739 | 44.44% (48/108) | 44.25% (5050/11412) | 0.968 |
| **Secondary outcome** | | | | | | |
| Positive β-hCG rate | 62.24% (122/196) | 61.99% (7085/11429) | 0.942 | 63.90% (69/108) | 62.00% (7138/11517) | 0.684 |
| Biochemical pregnancy rate | 8.67% (17/196) | 8.50% (971/11429) | 0.933 | 11.10% (12/108) | 8.48% (977/11517) | 0.330 |
| Implantation rate | 39.54% (138/349) | 40.00% (8195/20484) | 0.860 | 38.07% (75/197) | 40.02% (8258/20636) | 0.579 |
| Missing data on transfer outcome | 0.51% (1/196) | 0.91% (104/11429) | 0.557 | 0.00% (0/108) | 0.91% (105/11517) | 0.319 |
| Ectopic pregnancy rate | 1.94% (2/103) | 3.42% (203/5930) | 0.339 | 0.00% (0/57) | 3.43% (205/5976) | 0.155 |
| Miscarriage rate | 17.48% (18/103) | 13.46% (798/5930) | 0.237 | 14.04% (8/57) | 13.52% (808/5976) | 0.910 |
| Early miscarriage rate | 9.71% (10/103) | 9.39% (557/5930) | 0.913 | 12.28% (7/57) | 9.37% (560/5976) | 0.454 |
| Late miscarriage rate | 7.77% (8/103) | 4.06% (241/5930) | 0.061 | 1.75% (1/57) | 4.15% (248/5976) | 0.073 |
| Ongoing pregnancy rate | 46.94% (92/196) | 46.08% (5267/11429) | 0.815 | 46.30% (50/108) | 46.10% (5309/11517) | 0.970 |
| Preterm birth rate | 3.59% (7/195) | 8.96% (1015/11325) | 0.009 | 5.56% (6/108) | 8.90% (1016/11412) | 0.223 |
| Term birth rate | 39.49% (77/195) | 35.40% (4009/11325) | 0.237 | 39.81% (43/108) | 35.43% (4043/11412) | 0.343 |
