# Supplementary material for: Impact of spring festival on pregnancy outcomes in patients undergoing first embryo transfer: a retrospective cohort study
Source: Sci Rep. 2025 Jul 2;15:23339. doi: 10.1038/s41598-025-05029-6 (PMC12222965; doi:10.1038/s41598-025-05029-6)
Supplement: Supplementary file 2 — Supplementary Material 2 [file 41598_2025_5029_MOESM2_ESM.docx]

**Supplemental Material of “****Impact of Spring Festival on pregnancy outcomes in patients undergoing first embryo transfer: a retrospective cohort study”**


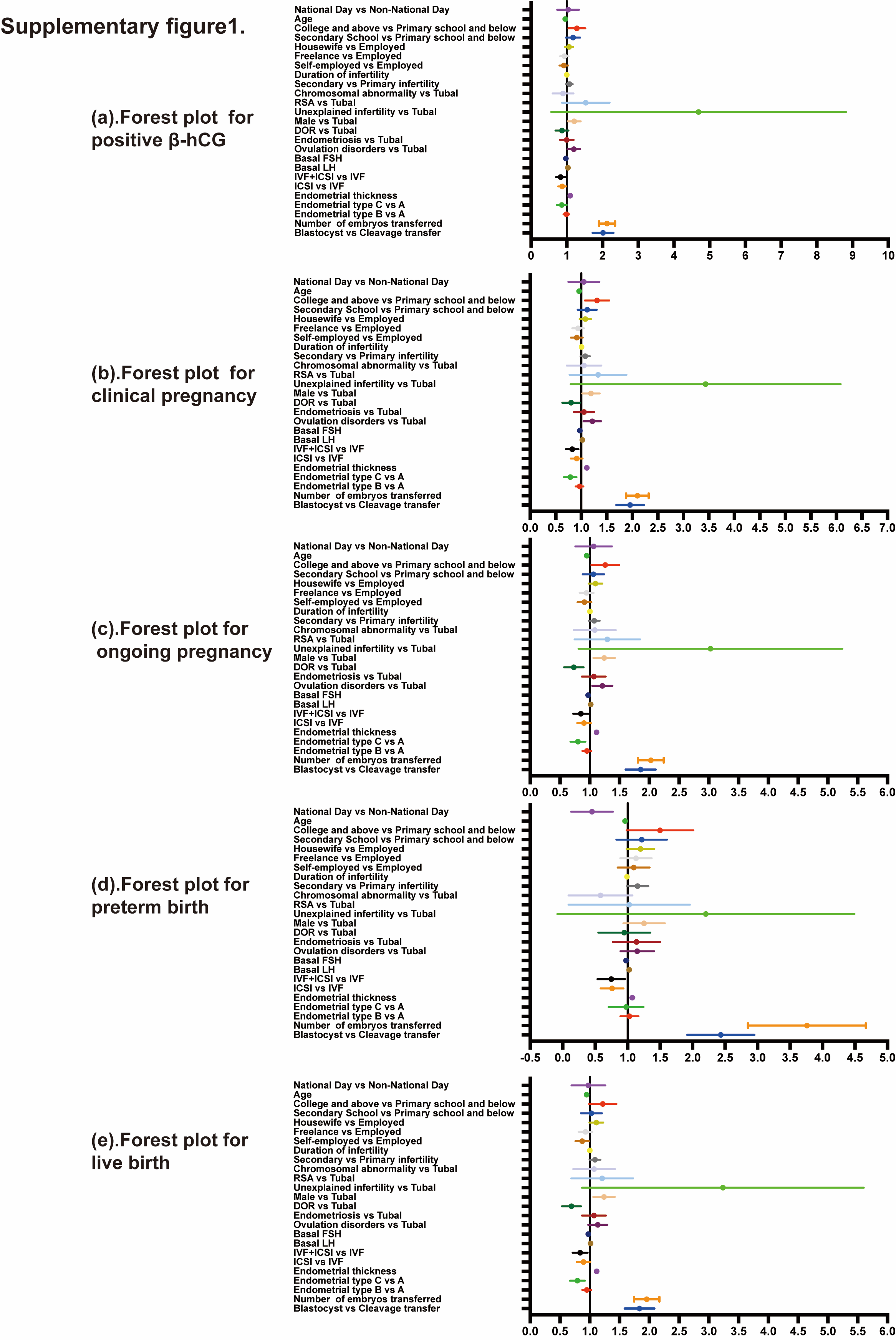


Supplementary Figure 1.Multivariate logistic regression analysis of clinical outcomes associated with embryo transfers performed during the National Day holiday period compared to routine periods. Adjusted odds ratios (aOR) with 95% confidence intervals are shown for key outcome measures (e.g.,positive-HCG, clinical pregnancy, ongoing pregnancy, preterm birth and live birth rate).


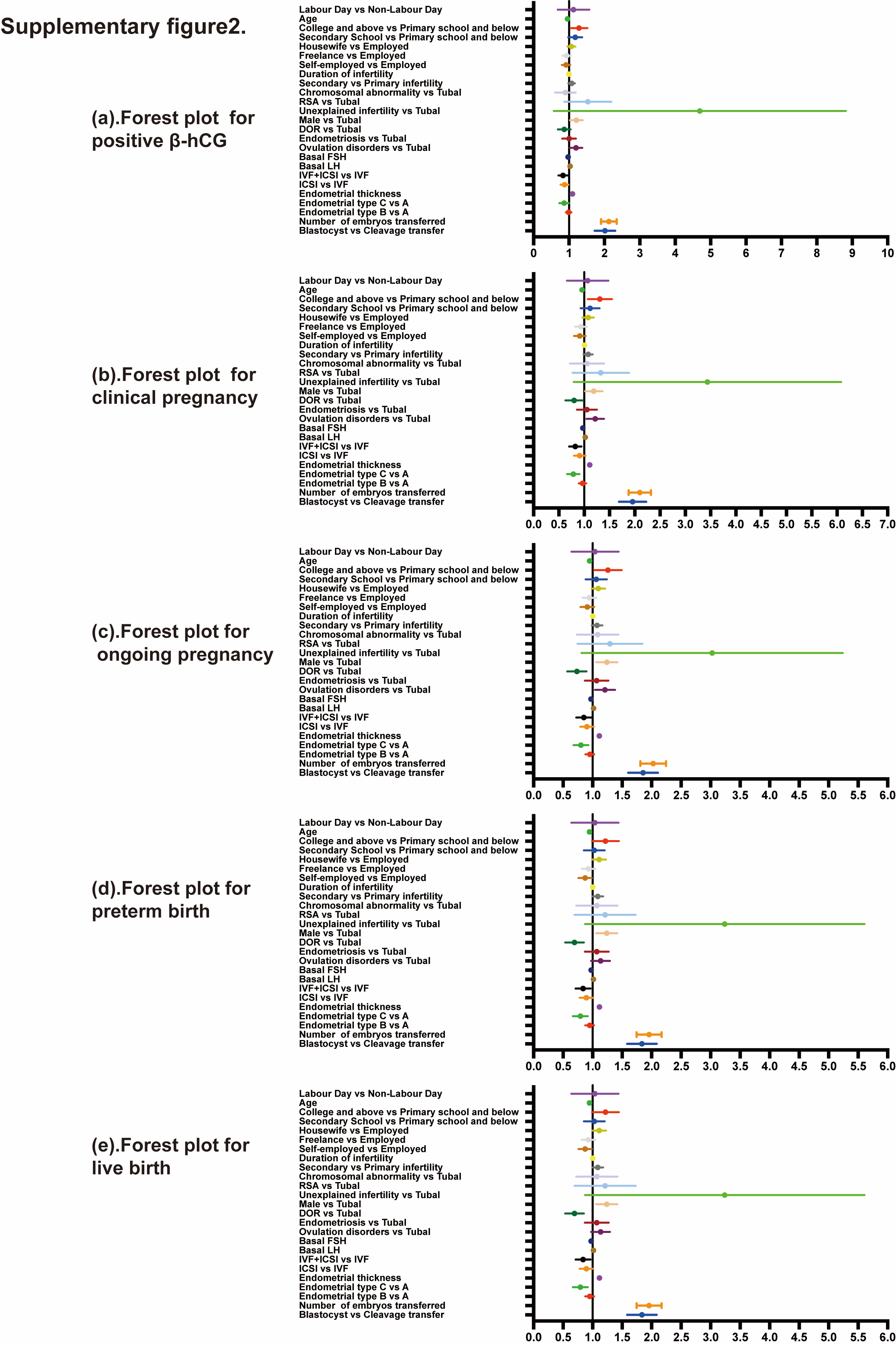


Supplementary Figure 2.Multivariate logistic regression analysis of clinical outcomes associated with embryo transfers performed during the Labour Day holiday period compared to routine periods. Adjusted odds ratios (aOR) with 95% confidence intervals are shown for key outcome measures (e.g.,positive-HCG, clinical pregnancy, ongoing pregnancy, preterm birth and live birth rate).
